# One health approach unravels worrying antimicrobial resistance patterns: A cross-sectional study in Kisii, Kenya

**DOI:** 10.1371/journal.pone.0331389

**Published:** 2025-09-03

**Authors:** Briton M. Kavulavu, Eric O. Omwenga, Oscar Asanya Nyangiri, Andrew K. Nyerere, Rael J. Too, Elizabeth J. Matey, Siri Göpel, Wycliffe Mogoa, Thorben Schilling, Ludwig E. Hoelzle, Beryl Primrose Gladstone

**Affiliations:** 1 Department of Medical Microbiology, Jomo Kenyatta University of Agriculture and Technology, Nairobi, Kenya; 2 Department of Medical Microbiology & Parasitology, School of Health Sciences, Kisii University, Kisii, Kenya; 3 Center for Microbiology Research, Kenya Medical Research Institute, Nairobi, Kenya; 4 German Center for Infection Research (DZIF), partner site Tübingen, Germany; 5 Department of Internal Medicine 1, University Hospital of Tübingen, Tübingen, Germany; 6 Department of Child Health and Pediatrics, School of Health Sciences, Kisii University, Kisii, Kenya; 7 Livestock Infectiology and Environmental Hygiene, University of Hohenheim, Stuttgart, Germany; Fayetteville State University, UNITED STATES OF AMERICA

## Abstract

**Background:**

Antimicrobial resistance (AMR) is a major public health challenge, particularly in Sub-Saharan Africa, where diagnostic and surveillance capacities are limited. *Enterobacterales* significantly contribute to AMR, with environmental reservoirs facilitating transmission between humans, animals, and the environment.

**Methods:**

This study investigated the prevalence and antimicrobial susceptibility of selected *Enterobacterales* in human, water, animal feces, and soil samples in Kenya. A cross-sectional study including 200 patients with gastrointestinal symptoms was conducted at Kisii Teaching and Referral Hospital and surrounding areas. AMR testing was performed using the disk diffusion method.

**Results:**

A total of 365 samples were collected: 200 human and 55 each of water, animal feces, and soil specimens from the homesteads of patients with resistant isolates. 343 isolates were obtained (*Escherichia coli*: 280/343 [81.6%], *Salmonella* spp.: 28/343 [8.2%], *Klebsiella* spp.: 25/343 [7.3%], *Shigella* spp.: 10/343 [2.9%]). A significant proportion of isolates exhibited AMR, particularly to piperacillin-tazobactam (up to 87%) and ampicillin (up to 79%). Resistance to piperacillin-tazobactam in *E. coli* was the highest, observed in humans (100/139, 71.9%), water (42/49, 85.7%), animal feces (9/46, 19.6%), and soil (33/46, 71.7%). Almost half (45%) of the human isolates showed ESBL production or resistance to imipenem, with water, animal feces, and soil samples, revealing similar resistance patterns. Resistance to chloramphenicol (71.7% vs 20.1%; p < 0.001) and third-generation cephalosporins were higher among animal and environmental isolates (animal feces: cefotaxime – 25/46, 54.3%; ceftazidime – 27/46, 58.7%) as compared to human isolates (cefotaxime – 40/139, 28.8%; ceftazidime – 28/139, 20.1%) (p < 0.001). In human isolates, the most prevalent genes were *blaTEM* (53/187, 28.3%), *blaOXA-48* (43/187, 23.0%), *blaSHV* (32/187, 17.1%), and *blaCTXM-15* (41/187, 21.9%); in animal isolates, *blaCTXM-8* (11/55, 20.0%), *blaVIM* (8/55, 14.5%), and *blaTEM* (8/55, 14.5%) were most detected; while in soil and water isolates, *blaCTXM-8* was the most common (10.9% and 9.1% respectively).

**Conclusion:**

Shared resistance patterns across human, animal, and environmental samples highlight interconnected AMR pathways. These findings reinforce the need for a One Health approach through integrated AMR surveillance and interventions.

## 1. Introduction

Antimicrobial resistance (AMR) has become one of the most significant public health threats globally. Resistant bacterial infections have been identified as a leading cause of mortality and morbidity. In 2019, nearly 5 million deaths were associated with AMR, of which 1.27 million were directly attributed to infections caused by resistant bacteria [1]. The World Health Organization (WHO) has repeatedly emphasised the urgency of controlling AMR and stated that without effective containment, the world risks returning to an era where common infections are untreatable [2]. The widespread misuse and overuse of antibiotics, both in healthcare and agriculture, have accelerated the rise of AMR across all sectors [3–5]. Bacteria belonging to the *Enterobacterales* family are of particular concern since they frequently cause serious infections and exhibit high resistance rates [6]. Thus, the global rise in AMR threatens essential therapeutic agents that were once effective but are becoming increasingly ineffective against common bacterial infections [7–9].

Sub-Saharan Africa (SSA) bears a disproportionate burden of AMR, a problem further compounded by resource-limited healthcare systems that lack robust diagnostic and surveillance capabilities [10–13]. In 2019, the WHO African region reported approximately 1.05 million deaths associated with bacterial AMR, with 250,000 of these being caused by resistant bacterial infections [14]. Diarrheal diseases, primarily due to infections in the gastrointestinal tract (GIT), are among the leading causes of death in children under five in SSA, and significant mortality occurs among adults in the region [15]. In SSA, diarrheal diseases account for approximately 194.5 deaths per 100,000 children under five and 33.5 deaths per 100,000 adults, underscoring their persistent burden across age groups [16,17]. These deaths are mostly caused by rotavirus or resistant bacterial pathogens [18]. Studies conducted in SSA demonstrate that resistance rates among *Enterobacterales* isolates are alarmingly high, with resistance to first-line [12,19] and even last-resort antibiotics increasingly documented both in clinical and environmental sources [12, 19–21]. This high burden of resistant infections creates significant challenges for healthcare providers, who often rely on empirical treatment due to limited access to laboratory facilities to conduct culture and susceptibility testing [22–24] ].

In Kenya, AMR among *Enterobacterales* is a pressing concern, particularly in rural areas where diagnostic facilities are often lacking or poorly equipped, limiting their ability to provide targeted treatment [12]. Resistance rates in Kenya mirror SSA, with recent studies estimating up to 67% of *E. coli* and 54% of *K. pneumoniae* hospital infections resistant to third-generation cephalosporins [19,21]. Gastrointestinal infections caused by these bacteria remain a leading concern in the region. One study documented a high prevalence of diarrheagenic *E. coli* at 36.4% among children, while other studies in rural Kenyan areas noted significant levels of *Shigella* spp. and *Salmonella* spp. among patients presenting with GIT symptoms [25]Despite the high prevalence of AMR in Kenya, research on local resistance patterns, particularly in referral hospitals away from major cities, remains limited despite these facilities serving enormous populations [26]. This problem is further complicated by the paucity of data from animal and environmental samples on AMR-related cases, more so, from the immediate environments where the interaction between animals, humans, and the general environment is real. This is very necessary as it can unravel the transmission dynamics of resistomes among the three core areas of One Health – human, animal and environment. Therefore, without such localized data, it will be hard to deduce the sources of such resistant strains and possibly influence treatment decisions on both humans and animals, thus relying on empirical choices, which are not always effective against resistant strains.

Acknowledging the interconnectedness of human, animal, and environmental health in addressing AMR and transmission possibilities across environmental reservoirs such as soil and water, as well as animals, for dissemination of antimicrobial resistance genes (ARGs) [27,28], a cross-sectional study was conducted at the Kisii Teaching and Referral Hospital (KTRH) to determine the prevalence and molecular epidemiology of *Enterobacterales* causing gastrointestinal infections. Patient and environment-related factors were also considered in relation to the resistant patterns to understand the epidemiology of transmission of resistant *Enterobacterales*.

## 2. Materials and methods

### 2.1 Study design and study area

A cross-sectional study was conducted from October 2023 to April 2024 among patients presenting with gastrointestinal infections at KTRH (1°16’07“S 36°58’45”E). KTRH is a 700-bed referral hospital located in Kisii County, Southwestern Kenya, which serves over five million people across the Counties of Kisii, Nyamira, Homa Bay, Migori, and Bomet. The study catchment area, is a highly populated area, and that comprises 60% rural and 40% semi-urban centres. Their major sources of income are agriculture and small-scale trade.

### 2.2 Study population and screening

Patients presenting with symptoms of GIT infections, including diarrhea, fever, vomiting, and mucoid or bloody stool, at the KTRH outpatient clinic between October 2023 and April 2024 were included in the study after obtaining written informed consent from them/their guardians. The sample size for this study was calculated using Fisher’s formula with a prevalence of 87% [29], resulting in a minimum sample size of 174 patients, which was then adjusted upwards to 200 patients for maximum coverage, factoring in samples with no isolates obtained. Selection of participants was by consecutive sampling. Stool samples were collected, and the antimicrobial susceptibility was tested for the isolated bacteria. Those exhibiting phenotypic resistance to extended-spectrum beta-lactamases (ESBLs) or carbapenem resistance (Imipenem) were followed up at their homes for additional animal and environmental sampling. Those without resistance to extended-spectrum beta-lactamases (ESBLs) or carbapenem resistance (Imipenem) were not followed up. One sample each of drinking water, animal feces of domestic animals, if available, and soil around the house was collected from the homesteads.

### 2.3 Sample collection, culture, and isolation

Stool samples were collected from patients and environmental sources, including animal feces, drinking water, and soil, using sterile containers and transported to the laboratory within two hours for processing as per previous protocols. Functional animals such as chickens and cows were selected owing to the farming practices of the locals, which involve manual handling of animal dung and usage as manure while still fresh. Transport of samples from the field to the laboratory was done using a cool box with ice packs inside to maintain the cold chain. Culturing involved using MacConkey and Salmonella-Shigella agar to isolate bacteria, with enrichment in Selenite F broth for enhanced recovery [30–35] (S1 File).

### 2.4 Antimicrobial susceptibility testing

Antimicrobial susceptibility testing (AST) was performed using the Kirby-Bauer disk diffusion method on Mueller-Hinton agar, following CLSI 2024 guidelines [36]. Isolates were tested against a range of antibiotics including; amoxicillin-clavulanate (AMC, 20/10 µg), ampicillin (AMP, 10 µg), trimethoprim-sulfamethoxazole (SXT, 1.25/23.75 µg), imipenem (IPM, 10 µg), cefotaxime (CTX, 30 µg), ceftazidime (CAZ, 30 µg), amikacin (AK, 30 µg), ceftriaxone (CRO, 30 µg), piperacillin-tazobactam (TZP, 100/10 µg), kanamycin (KAN, 30 µg), cefepime (FEP, 30 µg), chloramphenicol (CHL, 30 µg), and ciprofloxacin (CIP, 5 µg) (Oxoid, USA); and results were interpreted as susceptible, intermediate, or resistant based on breakpoints documented by CLSI 2024. The double-disk synergy method was used to detect extended-spectrum beta-lactamases (ESBLs) [37] (S1 File).

### 2.5 DNA extraction, PCR, and electrophoresis

DNA was extracted using the Invitrogen™ PureLink™ Genomic DNA Mini Kit following the manufacturer’s instructions, and the eluted DNA was stored at −20°C awaiting PCR. For PCR, a 25 μL reaction was prepared with 12.5 μL of Thermo Scientific™ DreamTaq PCR Master Mix (2X), 1 μL of each forward and reverse primer, 2 μL of DNA template, and 8.5 μL of nuclease-free water. The PCR cycle included initial denaturation at 94°C for 5 minutes, followed by 30 cycles of 95°C for 30 seconds, primer-specific annealing temperatures (Table 1), and 72°C extension, with a final extension at 72°C for 10 minutes. The study investigated ESBL (*blaTEM*, *blaSHV*, *blaCTX-M-1*, *blaCTX-M-9*, *blaCTX-M-8*, *blaCTX-M-15*) and carbapenemase-encoding (*blaVIM*, *blaNDM*, *blaKPC*, *blaIMP*, *blaOXA-48*) (Table 1). PCR products were separated on a 1% agarose tris-acetate-EDTA gel containing 0.5 μg of SYBR™ Safe DNA Gel Stain, using a 100–1,000 bp GeneRuler DNA Ladder to determine product size, and visualized under UV light (S1 File). The full study protocol is deposited at Protocols.io [41].

**Table 1:** List of primer sequences and their corresponding annealing temperatures and fragment sizes used for screening ESBL and carbapenemase-encoding genes.

Gene name	Primer sequence	Annealing Temp	Fragment size(bp)	Reference
blaTEM	F: AAACGCTGGTGAAAGTA R: AGCGATCTGTCTAT	46^o^C	822	[38]
blaSHV	F: ATGCGTTATATTCGCCTGTG R: TGCTTTGTTATTCGGGCCAA	53^o^C	753	
blaCTX-M-1	F: GGT TAA AAA ATC ACT GCG TC R: TTG GTG ACG ATT TTA GCC GC	55^o^C	850	
blaCTX-M – 9	F: ATG GTG ACA AAG AGA GTG CA R: CCC TTC GGC GAT GAT TCT C	53^o^C	850	
blaCTX-M − 8	F: TCGCGTTAAGCGGATGATGC R: AACCCACGATGTGGGTAG	57^o^C	666	
blaCTX-M-15	F: GTGATACCACTTCACCTC R: AGTAAGTGACCAGAATCAG	53^o^C	255	[39]
blaVIM	F: GATGGTGTTTGGTCGCATA R: CGAATGCGCAGCACCAG	53^o^C	390	[40]
blaNDM	F: GGTTTGGCGATCTGGTTTTC R: CGGAATGGCTCATCACGATC	53^o^C	621	
blaKPC	F: CGTCTAGTTCTGCTGTCTTG R: CTTGTCATCCTTGTTAGGCG	53^o^C	798	
blaIMP	F: GGAATAGAGTGGCTTAAYTCTC R: GGTTTAAYAAAACAACCACC	52^o^C	232	
blaOXA-48	F: GCGTGGTTAAGGATGAACAC R: CATCAAGTTCAACCCAACCG	53^o^C	438	

### 2.6 Quality control

Known control strains, including *E. coli* ATCC 25922 and *Klebsiella pneumoniae* ATCC 700603, were used to verify the accuracy of biochemical tests and antimicrobial susceptibility testing results [4].

### 2.7 Data analysis and presentation

Data from paper records were initially entered into dedicated databases, cleaned, and validated. All statistical analyses were conducted using Stata version 15 (StataCorp LLC). Descriptive statistics, including frequencies and percentages, were used to summarize demographic characteristics, prevalence, susceptibility profiles, and frequencies of ESBL and carbapenemase encoding genes. Association of resistance patterns of isolated pathogens with the sample types was studied using Chi-square statistic, with only the statistically significant associations being reported in this manuscript. Visualisation of the geographical distribution of samples was facilitated using village coordinates based on Google Earth 7.1 and QGIS 3.30 [42,43].

### 2.8 Ethical considerations

Ethical approval for this study was obtained from the Moi University-Moi Teaching and Referral Hospital Institutional Review and Ethics Committee (MU-MTRH IREC) (Approval No: 0004012) and the National Commission for Science, Technology and Innovation (NACOSTI) (Approval No: NC44894). Written informed consent was obtained for adults, written assent with parental consent for participants aged 12–17 years, and written parental consent for those below 12 years using standardized informed consent forms (S2 File). Individuals who refused to consent or assent were excluded from the study. Confidentiality and anonymity were maintained for all collected data. Clinically relevant results were shared with patients’ healthcare providers for appropriate follow-up.

## 3. Results

### 3.1 Study population and patient characteristics

Overall, 200 patients with suspected GI infection were included in the study. The median age was 34 years (IQR: 22 years), with 3% being below 5 years of age. They were predominantly females (n = 116, 58%), had attained secondary (n = 83, 41.5%) or tertiary (n = 81, 40.5%) level education, and commonly had farming (n = 49, 24.5%) and business (n = 35, 17.5%) as occupations. The majority of participants in the study had no animal exposure (132, 66.0%), referring to any form of interactions with animals, such as keeping, working with, or being in contact with animal products or waste. On the contrary, 34.0% (n = 68) reported having at least a form of animal exposure. Most sourced their meat from markets (198, 99%), with only 17.5% (35) relying on their own animals. Most participants obtained vegetables from markets (184, 92%), and common water sources included treated piped water (101, 50.5%) and underground wells (73, 36.5%). While 32% (n = 64) kept domestic animals, 32.5% (n = 65) worked with animals, with 11.5% (n = 23) reporting antibiotic use on animals. Prior unprescribed antibiotic usage within the previous 2 weeks was reported by 18 patients (9%), while the remainder had not used any medication (Table 2).

**Table 2:** Demographic and clinical characteristics of patients included in the study (N = 200).

Patient characteristic	Categories	Frequency (%)
**Sex**	Female	116 (58.0)
	Male	84 (42.0)
**Education**	Primary	26 (13.0)
	Secondary	83 (41.5)
	Tertiary	81 (40.5)
	None	10 (5.0)
**Occupation**	Students/Pupils	57 (28.5)
	Farming	49 (24.5)
	Business	35 (17.5)
	Teachers	28 (14.0)
	Healthcare	14 (7.0)
	Unemployed	6 (3.0)
	Manual Laborers	3 (1.5)
	Others	6 (3.0)
**Age group**	Below 5 years	6 (3.0)
	5-14 years	16 (8.0)
	15-24 years	37 (18.5)
	25-44 years	97 (48.5)
	45-59 years	26 (13.0)
	60 years and above	18 (9.0)
**Presenting symptoms**	Diarrhea	200 (100.0)
	Fever	182 (91.0)
	Vomiting	106 (53.0)
	Mucoid/Bloody stool	105 (52.5)
**Source of meat**	Market	198 (99)
	Own Animal	35 (17.5)
	Not Applicable	2 (1)
**Water sources**	Treated Piped Water	101 (50.5)
	Underground Well	73 (36.5)
	Rain Water	25 (12.5)
	Springs	37 (18.5)
	River	48 (24)
	Other (water vendors)	1 (0.5)
**Exposure to animals** ^*^	Any form of animal exposure	68 (34.0)
	Pets in the homestead	40 (20)
	Keeps domestic animals	64 (32)
	Works with animals	65 (32.5)
**Prior unprescribed antibiotic usage**	Yes	18 (9.0)
	No	182 (91.0)
**Type of animal contact at work**	Feeding and Cleaning	55 (27.5)
	Processing Meat	13 (6.5)
**Antibiotic usage in animals**	Yes	23 (11.5)
	No	177 (89.5)

* Exposure to animals categories: “Any form of animal exposure” refers to interaction with animals in any capacity; “Pets in the homestead: refers to cats and dogs kept for companionship; “Keeps domestic animals” refers to cows, goats, sheep, pigs, and chickens kept for functional purposes such as farming; and “Works with animals” refers to being engaged in animal-related occupations.

### 3.2 Bacterial isolates

**Fig 1:**
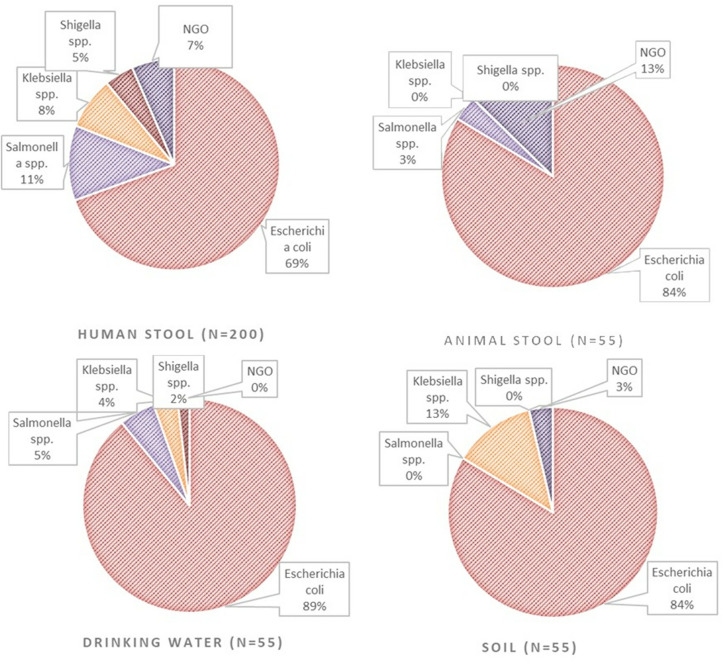
Frequency distribution of *Enterobacterales* species isolated from samples tested. A total of 365 samples were collected, including 200 human stool, and 55 each of animal feces, water and soil samples. The most common isolate was *E. coli*. *Note: (NGO) no growth obtained.*

**Fig 2:**
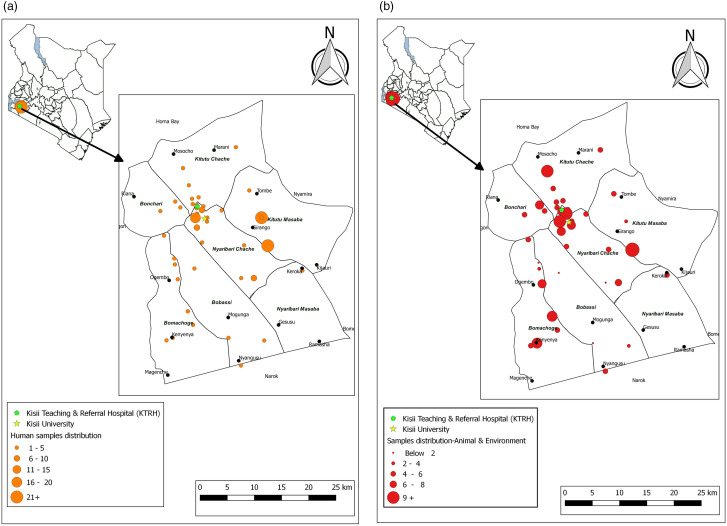
Geographical distribution of the study samples. (a) is a representation of the distribution of the patient samples while (b) shows distribution of the sources of animal and environmental samples (b) across Kisii County. [Prepared using custom coordinates and study data in Q-GIS 3.30].

The 200 human stool samples analyzed yielded 187 isolates. The prevalences were as follows: *E. coli* (n = 139, 69.5%), *Salmonella* spp. (n = 23, 11.5%), *Klebsiella* spp. (n = 16, 8%), and *Shigella* spp*.* (n = 9, 4.5%). A total of 13 (6.5%) samples yielded no growth from among the human stool samples collected. ESBL production and/or resistance to imipenem was detected in 84/187 (44.9%) isolated pathogens. Whereas all the 84 met the criteria for environmental and animal sampling at the homesteads, animal and environmental samples were only collected from the household surroundings of 24 patients. The rest were cases of unsuccessful follow-up, either due to participants being unreachable via the telephone numbers they provided or not consenting to be visited at their homesteads. To bridge this gap, the locations of these patients were aggregated, and samples were taken from public places in the same locality for an additional 31 sites, with one sample of drinking water, animal feces, and soil being collected from each site. Shared water points, fresh animal feces around water points, and soil in similar areas were collected as per the described procedures. Similar to the human samples, *E. coli* was the most common organism in animal and environmental samples, having been detected in drinking water (n = 49/55, 89.1%), animal feces (n = 46/55, 83.6%), and soil (n = 46/55, 83.6%), followed by *Salmonella* spp., *Klebsiella* spp., and *Shigella* spp. as in Fig 1. Overall, 343 isolates were obtained from the 365 samples (*E. coli*: 280/343 [81.6%], *Salmonella* spp.: 28/343 [8.2%], *Klebsiella* spp.: 25/343 [7.3%], *Shigella* spp.: 10/343 [2.9%]). The distribution of the isolates from these samples across all sampled locations is shown in Fig 2.

### 3.3 Antimicrobial resistance profiles

**Fig 3:**
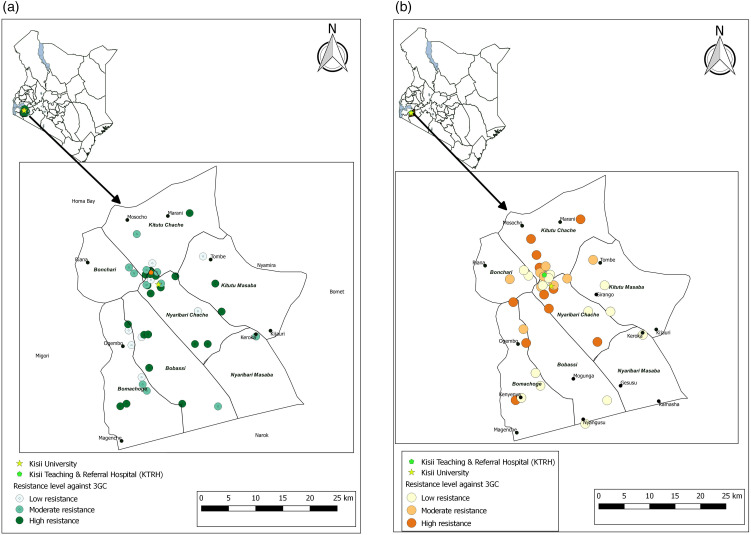
Geographical distribution of resistance to third-generation cephalosporins across Kisii County. The distribution of human samples with *Enterobacterales* resistant to third-generation cephalosporins **(a)**, with the corresponding distribution for animal and environmental samples **(b)** [Prepared using custom coordinates and study data in Q-GIS 3.30].

**Fig 4:**
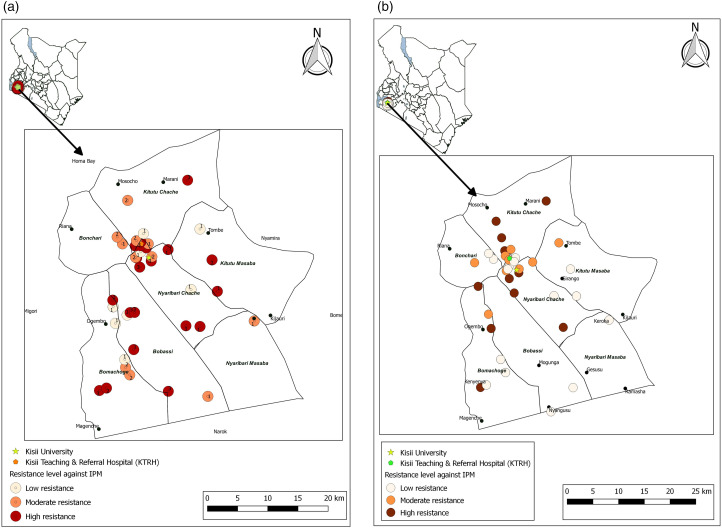
Geographical distribution of resistance to imipenem across Kisii County. The distribution of human samples with *Enterobacterales* resistant to imipenem **(a)**, with the corresponding distribution for animal and environmental samples **(b)** [Prepared using custom coordinates and study data in Q-GIS 3.30].

The antimicrobial susceptibility profiles demonstrated significant variability across isolates from human stool. The highest sensitivity was observed for ceftazidime in *E. coli* (105/139, 75.5%), *Salmonella* spp. (18/23, 78.3%), *Klebsiella* spp. (11/16, 68.8%), and *Shigella* spp. (7/9, 77.8%), and for cefotaxime in *E. coli* (92/139, 66.2%), *Salmonella* spp. (15/23, 65.2%), and *Klebsiella* spp. (10/16, 62.5%). Conversely, resistance was most notable for ampicillin in *E. coli* (109/139, 78.4%), *Klebsiella* spp. (12/16, 75.0%), and *Shigella* spp. (7/9, 77.8%) and for trimethoprim-sulfamethoxazole in *E. coli* (82/139, 59.0%) and *Shigella* spp. (7/9, 77.8%). Similarly, significant resistance was observed for piperacillin-tazobactam in *E. coli* (100/139, 71.9%), *Klebsiella* spp. (13/16, 81.3%), and *Shigella* spp. (7/9, 77.8%) (Table 3). The geographical distribution of the resistance patterns of isolates from human stools resistant to third-generation cephalosporins (3GC) (Fig 3(a)) and Imipenem (IPM) (Fig 4(a)) among the human stool did not show any tendency towards clustering.

**Table 3:** Antimicrobial resistance profiles of isolates from human stool samples per species (N = 187).

Organism	Suscep-tibility	Antibiotics [n (%)]												
		AMC	AMP	SXT	IPM	CTX	CAZ	AK	CRO	TZP	KAN	FEP	CHL	CIP
*Escherichia coli (n = 139)*	S	43 (30.9)	23 (16.6)	49 (35.3)	64 (46.0)	92 (66.2)	105 (75.5)	90 (64.8)	68 (48.9)	22 (15.8)	38 (27.3)	88 (63.3)	92 (66.2)	80 (57.6)
	I	17 (12.2)	7 (5.0)	8 (5.8)	46 (33.1)	7 (5.0)	6 (4.3)	15 (10.8)	36 (25.9)	17 (12.2)	63 (45.3)	8 (5.8)	3 (2.2)	12 (8.6)
	R	78 (56.1)	109 (78.4)	82 (59.0)	29 (20.8)	40 (28.8)	28 (20.1)	34 (24.5)	35 (25.3)	100 (71.9)	38 (27.3)	43 (30.9)	44 (31.6)	47 (33.8)
*Salmonella* spp. *(n = 23)*	S	3 (13.0)	10 (43.5)	5 (21.7)	8 (34.9)	15 (65.2)	18 (78.3)	16 (69.6)	10 (43.5)	4 (17.4)	9 (39.1)	14 (60.9)	11 (47.8)	12 (52.2)
	I	2 (8.7)	--	3 (13.0)	4 (17.4)	2 (8.7)	2 (8.7)	3 (13.0)	5 (21.7)	2 (8.7)	10 (43.5)	3 (13.0)	2 (8.7)	3 (13.0)
	R	17 (73.9)	12 (52.2)	15 (65.2)	11 (47.8)	6 (26.1)	3 (13.0)	4 (17.4)	8 (34.8)	17 (73.9)	4 (17.4)	6 (26.1)	10 (43.5)	8 (34.8)
*Klebsiella* spp. *(n = 16)*	S	7 (43.8)	3 (18.8)	3 (18.8)	9 (56.3)	10 (62.5)	11 (68.8)	8 (50.0)	6 (37.5)	2 (12.5)	4 (25.0)	8 (50.0)	7 (43.8)	8 (50.0)
	I	3 (18.8)	--	1 (6.3)	2 (12.5)	1 (6.3)	--	2 (12.5)	4 (25.0)	1 (6.3)	6 (37.5)	1 (6.3)	--	1 (6.3)
	R	5 (31.3)	12 (75.0)	11 (68.8)	5 (31.3)	5 (31.3)	5 (31.3)	6 (37.5)	6 (37.5)	13 (81.3)	6 (37.5)	7 (43.8)	9 (56.3)	7 (43.8)
*Shigella* spp.*(n = 9)*	S	5 (55.6)	2 (22.2)	2 (22.2)	4 (44.4)	5 (55.6)	7 (77.8)	6 (66.7)	4 (44.4)	1 (11.1)	3 (33.3)	5 (55.6)	5 (55.6)	5 (55.6)
	I	2 (22.2)	--	--	2 (22.2)	--	1 (11.1)	1 (11.1)	2 (22.2)	1 (11.1)	4 (44.4)	2 (22.2)	1 (11.1)	1 (11.1)
	R	2 (22.2)	7 (77.8)	7 (77.8)	3 (33.3)	4 (44.4)	1 (11.1)	2 (22.2)	3 (33.3)	7 (77.9)	2 (22.2)	2 (22.2)	3 (33.3)	3 (33.3)

S: Sensitive; I: Intermediate, R: Resistant; AMC: Amoxicillin-Clavulanate; AMP: Ampicillin; SXT: Trimethoprim-Sulfamethoxazole; IPM: Imipenem; CTX: Cefotaxime; CAZ: Ceftazidime; AK: Amikacin; CRO: Ceftriaxone; TZP: Piperacillin-Tazobactam; KAN: Kanamycin; FEP: Cefepime; CHL: Chloramphenicol; CIP: Ciprofloxacin

--: No isolate

Among isolates from the drinking water, *E. coli* (n = 49) showed high sensitivity to ciprofloxacin (39/49, 79.6%) and notably lower sensitivity to piperacillin-tazobactam (7/49, 14.3%), *Salmonella* spp. (n = 3) exhibited complete sensitivity to amikacin and amoxicillin-clavulanate 3/3 (100%), *Klebsiella* spp. (n = 2) showed partial sensitivity to ciprofloxacin 1/2 (50%), and *Shigella* spp. (n = 1) was fully sensitive to amikacin 1/1 (100%) (Table 4).

**Table 4:** Antimicrobial resistance profiles of isolates from drinking water samples per species (N = 55).

Organism	Susceptibility	Antibiotics [n (%)]												
		AMC	AMP	SXT	IPM	CTX	CAZ	AK	CRO	TZP	KAN	FEP	CHL	CIP
*Escherichia coli (n = 49)*	S	21 (42.9)	24 (49.0)	17 (34.7)	11 (22.4)	28 (57.1)	36 (73.5)	21 (42.9)	14 (28.6)	--	15 (30.6)	23 (46.9)	35 (71.4)	39 (79.6)
	I	10 (20.4)	1 (2.0)	--	10 (20.4)	9 (18.4)	1 (2.0)	11 (22.4)	9 (18.4)	7 (14.3)	25 (51.0)	4 (8.2)	3 (6.1)	4 (8.2)
	R	18 (36.7)	24 (49.0)	32 (65.3)	28 (57.1)	12 (24.5)	12 (24.5)	17 (34.7)	26 (53.1)	42 (85.7)	9 (18.4)	22 (44.9)	11 (22.4)	6 (12.2)
*Salmonella* spp.*(n = 3)*	S	3 (100.0)	3 (100.0)	2 (66.7)	--	1 (33.3)	1 (33.3)	3 (100.0)	1 (33.3)	--	2 (66.7)	1 (33.3)	2 (66.7)	3 (100.0)
	I	--	--	--	1 (33.3)	2 (66.7)	--	--	--	2 (66.7)	1 (33.3)	--	--	--
	R	--	--	1 (33.3)	2 (66.7)	--	2 (66.7)	--	2 (66.7)	1 (33.3)	--	2 (66.7)	1 (33.3)	--
*Klebsiella* spp.*(n = 2)*	S	--	1 (50.0)	--	1 (50.0)	1 (50.0)	1 (50.0)	--	2 (100.0)	--	--	--	2 (100.0)	1 (50.0)
	I	1 (50.0)	--	--	--	--	--	2 (100.0)	--	--	2 (100.0)	2 (100.0)	--	1 (50.0)
	R	1 (50.0)	1 (50.0)	2 (100.0)	1 (50.0)	1 (50.0)	1 (50.0)	--	--	2 (100.0)	--	--	--	--
*Shigella* spp. *(n = 1)*	S	1 (100.0)	--	1 (100.0)	--	--	--	1 (100.0)	--	--	--	--	1 (100.0)	1 (100.0)
	I	--	--	--	--	--	--	--	--	--	--	--	--	--
	R	--	1 (100.0)	--	1 (100.0)	1 (100.0)	1 (100.0)	--	1 (100.0)	1 (100.0)	1 (100.0)	1 (100.0)	--	--

S: Sensitive; I: Intermediate, R: Resistant; AMC: Amoxicillin-Clavulanate; AMP: Ampicillin; SXT: Trimethoprim-Sulfamethoxazole; IPM: Imipenem; CTX: Cefotaxime; CAZ: Ceftazidime; AK: Amikacin; CRO: Ceftriaxone; TZP: Piperacillin-Tazobactam; KAN: Kanamycin; FEP: Cefepime; CHL: Chloramphenicol; CIP: Ciprofloxacin

--: No isolate

In animal feces isolates, *E. coli* (46/55, 83.6%) showed notable resistance to ampicillin (33/46, 71.7%) and trimethoprim-sulfamethoxazole (31/46, 67.4%). Resistance to ceftazidime was recorded at 27/46 (58.7%). *Salmonella* spp. isolates (2/55, 3.6%) exhibited resistance to ampicillin, cefotaxime, and trimethoprim-sulfamethoxazole (2/2, 100%) (Table 5).

**Table 5:** Antimicrobial resistance profiles of isolates from animal stool sample per species (N = 48).

Organism	Susceptibility	Antibiotics [n (%)]												
		AMC	AMP	SXT	IPM	CTX	CAZ	AK	CRO	TZP	KAN	FEP	CHL	CIP
*Escherichia coli (n = 46)*	S	16 (34.8)	12 (26.1)	14 (30.4)	24 (52.2)	15 (32.6)	17 (37.0)	42 (91.3)	17 (37.0)	23 (50.0)	22 (47.8)	22 (47.8)	35 (76.1)	37 (80.4)
	I	7 (15.2)	1 (2.2)	1 (2.2)	11 (23.9)	6 (13.0)	2 (4.3)	4 (8.7)	3 (6.5)	14 (30.4)	9 (19.6)	6 (13.0)	6 (13.0)	5 (10.9)
	R	23 (50.0)	33 (71.7)	31 (67.4)	11 (23.9)	25 (54.3)	27 (58.7)	--	26 (56.5)	9 (19.6)	15 (32.6)	18 (39.1)	5 (10.9)	4 (8.7)
*Salmonella* spp. (n = 2)	S	2 (100)	--	--	--	--	--	2 (100)	--	2 (100)	1 (50)	2 (100)	2 (100)	1 (50)
	I	--	--	--	2 (100)	--	--	--	--	--	--	--	--	--
	R	--	2 (100)	2 (100)	--	2 (100)	2 (100)	--	2 (100)	--	1 (50)	--	--	1 (50)

S: Sensitive; I: Intermediate, R: Resistant; AMC: Amoxicillin-Clavulanate; AMP: Ampicillin; SXT: Trimethoprim-Sulfamethoxazole; IPM: Imipenem; CTX: Cefotaxime; CAZ: Ceftazidime; AK: Amikacin; CRO: Ceftriaxone; TZP: Piperacillin-Tazobactam; KAN: Kanamycin; FEP: Cefepime; CHL: Chloramphenicol; CIP: Ciprofloxacin

--: No isolate

Finally, for the isolates from soil, *E. coli* (46/55, 83.6%) exhibited resistance to ampicillin (29/46, 63%) and trimethoprim-sulfamethoxazole (24/46, 52.2%). Resistance to amoxicillin-clavulanate was observed in 27/46 (58.7%). Among *Klebsiella* spp. isolates (7/55, 12.7%), resistance to trimethoprim-sulfamethoxazole (4/7, 57.1%) and ampicillin (3/7, 42.9%) was notable (Table 6). The geographical distribution of the resistance patterns of isolates from animal and environmental sources resistant to third-generation cephalosporins (3GC) (Fig 3(b)) and Imipenem (IPM) (Fig 4(b)) among the human stool did not show any tendency towards clustering.

**Table 6:** Antimicrobial resistance profiles of isolates from soil samples per species (N = 53).

Organism	Susceptibility	Antibiotics [n (%)]												
		AMC	AMP	SXT	IPM	CTX	CAZ	AK	CRO	TZP	KAN	FEP	CHL	CIP
*Escherichia coli (n = 46)*	S	17 (37.0)	15 (32.6)	21 (45.7)	11 (23.9)	20 (43.5)	27 (58.7)	42 (91.3)	36 (78.3)	13 (28.3)	38 (82.6)	41 (89.1)	13 (28.3)	36 (78.3)
	I	2 (4.3)	2 (4.3)	1 (2.2)	24 (52.2)	13 (28.3)	5 (10.9)	--	--	--	--	--	--	6 (13.0)
	R	27 (58.7)	29 (63.0)	24 (52.2)	11 (23.9)	13 (28.3)	14 (30.4)	4 (8.7)	10 (21.7)	33 (71.7)	8 (17.4)	5 (10.9)	33 (71.7)	4 (8.7)
*Klebsiella* spp. *(n = 7)*	S	5 (71.4)	4 (57.1)	3 (42.9)	3 (42.9)	4 (57.1)	6 (85.7)	7 (100)	3 (42.9)	2 (28.6)	6 (85.7)	6 (85.7)	3 (42.9)	7 (100)
	I	1 (14.3)	--	--	4 (57.1)	1 (14.3)	1 (14.3)	--	--	--	--	--	--	--
	R	1 (14.3)	3 (42.9)	4 (57.1)	--	2 (28.6)	--	--	4 (57.1)	5 (71.4)	1 (14.3)	1 (14.3)	4 (57.1)	--

S: Sensitive; I: Intermediate, R: Resistant; AMC: Amoxicillin-Clavulanate; AMP: Ampicillin; SXT: Trimethoprim-Sulfamethoxazole; IPM: Imipenem; CTX: Cefotaxime; CAZ: Ceftazidime; AK: Amikacin; CRO: Ceftriaxone; TZP: Piperacillin-Tazobactam; KAN: Kanamycin; FEP: Cefepime; CHL: Chloramphenicol; CIP: Ciprofloxacin

--: No isolate

### 3.4 Comparing resistance patterns across human, animal, and environmental samples

**Fig 5:**
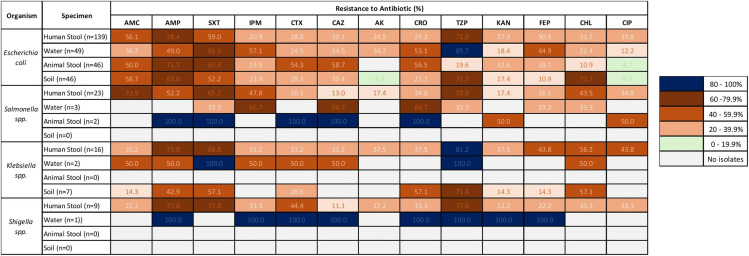
Resistance rates among *Enterobacterales* isolates from human, animal, and environmental samples. The percentage refer to percentage of resistant isolates to the total isolates. Abbreviations – AMC: Amoxicillin-Clavulanate; AMP: Ampicillin; SXT: Trimethoprim-Sulfamethoxazole; IPM: Imipenem; CTX: Cefotaxime; CAZ: Ceftazidime; AK: Amikacin; CRO: Ceftriaxone; TZP: Piperacillin-Tazobactam; KAN: Kanamycin; FEP: Cefepime; CHL: Chloramphenicol; CIP: Ciprofloxacin. Chi-square tests revealed significant associations for piperacillin-tazobactam, chloramphenicol, and third-generation cephalosporins (p < 0.05) only. Other associations lacked statistical significance.

The AMR profiles demonstrated overlapping resistance patterns across human and environmental isolates. Resistance to ampicillin, amoxicillin-clavulanate and sulfamethoxazole-trimethoprim was generally high among *E. coli* isolates across human, animal, and environmental samples. Resistance to piperacillin-tazobactam in *Escherichia coli* detected in human stool (100/139, 71.9%) was comparably high as resistance rates in *E. coli* isolated from water (42/49, 85.7%), and soil (33/46, 71.7%) samples; however, *E. coli* isolates from animal feces samples (9/46, 19.6%) exhibited lower resistance (p < 0.001). Interestingly, resistance to chloramphenicol was higher in *E. coli* isolated from the soil samples as compared to human stool (71.7% vs 20.1%; p < 0.001). Similarly, resistance rate to any of the third-generation cephalosporins was higher in *E. coli* isolates from animal and water samples (89.1% and 77.6%) than human *E. coli* isolates (57.6%, p < 0.001). Resistance rates of isolates by type of sample is provided in Fig 5.

**Fig 6:**
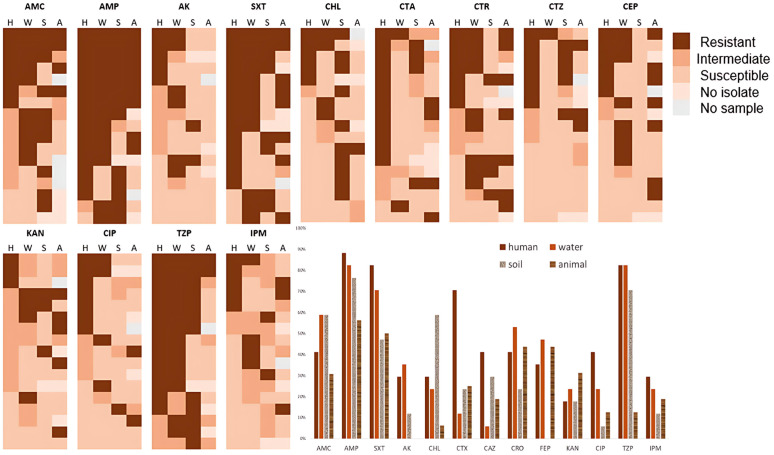
Susceptibility pattern of *E. coli* isolates from human stool (H) alongside water (W), soil (S) and animal stool (A) samples collected from the home environment of the patients (n = 17). Each row refers to the different samples related to the same patient. The bar graph shows the resistance rates among the samples. Abbreviations – AMC: Amoxicillin-Clavulanate; AMP: Ampicillin; SXT: Trimethoprim-Sulfamethoxazole; IPM: Imipenem; CTX: Cefotaxime; CAZ: Ceftazidime; AK: Amikacin; CRO: Ceftriaxone; TZP: Piperacillin-Tazobactam; KAN: Kanamycin; CEP: Cefepime; CHL: Chloramphenicol; CIP: Ciprofloxacin.

Of 24 patients for whom animal and environmental samples were collected at their homes, *E. coli* isolates were detected in both human, animal, and environmental samples in 17 (70.8%) patients. The susceptibility pattern of *E. coli* isolates was compared within the human-animal-environmental samples. Generally, *E. coli* isolates from the animal and environmental samples had a higher probability of exhibiting resistance when the human isolate was resistant; the association was only significant for water and animal feces samples (p < 0.01). The susceptibility patterns of the pairs of *E. coli* isolates from the human and environmental samples are presented in Fig 6.

### 3.5 Frequency and distribution of ESBL and CRB Genes

The study revealed the notable prevalence of both ESBL and CRB genes across different sample types. *blaTEM* was the most prevalent ESBL gene, particularly in human stool (28.3%) and animal feces (16.7%). In contrast, *blaKPC* was the most common CRB gene, with the highest occurrence in animal stool (14.6%) and human stool (7.5%). The *blaIMP* was absent across all samples. Identification of similar ARGs across the samples points to possible multidrug resistance, as shown in Table 7. Similarly, no significant association was observed between the genes and organisms at the p < 0.05 level (Table 8).

**Table 7:** Frequency of ESBL and CRB genes detected in the sample isolates from human, animal, and environmental samples collected during the study (N = 343).

Name of the gene detected	Human Stool (%) (n = 187)	Drinking Water (%) (n = 55)	Animal Stool (%) (n = 48)	Soil (%) (n = 53)	Total (%) (N = 343)
*blaKPC*	14 (7.5)	1 (1.8)	7 (14.6)	3 (5.7)	25 (7.3)
*blaNDM*	28 (15.0)	1 (1.8)	3 (6.3)	4 (7.5)	36 (10.5)
*blaVIM*	24 (12.8)	3 (5.5)	8 (16.7)	3 (5.7)	38 (11.1)
*blaOXA-48*	43 (23.0)	4 (7.3)	6 (12.5)	3 (5.7)	56 (16.3)
*blaIMP*	0 (0.0)	0 (0.0)	0 (0.0)	0 (0.0)	0 (0.0)
*blaSHV*	32 (17.1)	3 (5.5)	3 (6.3)	2 (3.8)	40 (11.7)
*blaTEM*	53 (28.3)	4 (7.3)	8 (16.7)	4 (7.5)	69 (20.1)
*blaCTXM-1*	15 (8.0)	0 (0.0)	1 (2.1)	0 (0.0)	16 (4.7)
*blaCTXM-9*	8 (4.3)	0 (0.0)	2 (4.2)	0 (0.0)	10 (2.9)
*blaCTXM-8*	24 (12.8)	5 (9.1)	11 (22.9)	6 (11.3)	46 (13.4)
*blaCTXM-15*	41 (21.9)	2 (3.6)	5 (10.4)	1 (1.9)	49 (14.3)

**Table 8:** Frequency of ESBL and CRB genes detected per organism (N = 343).

Name of the gene detected	*Escherichia coli* (n = 280) (%)	*Klebsiella* spp. (n = 25) (%)	*Salmonella* spp. (n = 28) (%)	*Shigella* spp. (n = 10) (%)	Total (N = 343) (%)	P value
***blaKPC***	22 (7.86)	0 (0.00)	2 (7.14)	1 (10.00)	25 (7.29)	0.637
***blaNDM***	27 (9.64)	5 (20.00)	2 (7.14)	2 (20.00)	36 (10.50)	0.173
***blaVIM***	29 (10.36)	4 (16.00)	4 (14.29)	1 (10.00)	38 (11.08)	0.874
***blaOXA-48***	45 (16.07)	5 (20.00)	4 (14.29)	2 (20.00)	56 (16.33)	0.681
***blaIMP***	0 (0.00)	0 (0.00)	0 (0.00)	0 (0.00)	0 (0.00)	--
***blaSHV***	32 (11.43)	4 (16.00)	4 (14.29)	1 (10.00)	40 (11.66)	0.929
***blaTEM***	52 (18.57)	9 (36.00)	6 (21.43)	2 (20.00)	69 (20.12)	0.201
***blaCTXM-1***	13 (4.64)	1 (4.00)	2 (7.14)	0 (0.00)	16 (4.66)	0.922
***blaCTXM-9***	10 (3.57)	0 (0.00)	0 (0.00)	0 (0.00)	10 (2.92)	0.718
***blaCTXM-8***	37 (13.21)	4 (16.00)	3 (10.71)	2 (20.00)	46 (13.41)	0.567
***blaCTXM-15***	38 (13.57)	5 (20.00)	5 (17.86)	1 (10.00)	49 (14.26)	0.697

Note: This table shows the detection frequencies of each gene across different organisms, their corresponding percentages, and the **P value** indicating the statistical association between each gene and the respective organism. No statistically significant association was observed.

## 4. Discussion

Our cross-sectional study among patients presenting with gastrointestinal symptoms at a referral hospital in Southwestern Kenya and their animals and environment found that *E. coli* was the most prevalent pathogen in both human stool, animal feces, and environmental samples, followed by *Salmonella* spp.*, Klebsiella* spp., and *Shigella* spp. A significant proportion of these isolates exhibited notable levels of AMR, particularly to piperacillin-tazobactam and ampicillin. Almost half of the human isolates showed ESBL production or resistance to imipenem, with the animal feces, water, and soil samples, revealing similar resistance patterns. Resistance to chloramphenicol and third-generation cephalosporins was higher among animal and environmental isolates than human isolates. The study also detected a high prevalence of ESBL and CRB genes.

Our results identified *E. coli* as the predominant organism across human, animal, and environmental samples, which is consistent with previous literature. Studies show that *E. coli* has been documented to be the number one contaminant of water sources in SSA, an indicator of fecal water contamination [44,45]. Its presence in water, soil, and animal feces samples further highlights its environmental adaptability and the potential interconnected transmission between humans, animals, and the environment [46,47]. Similarly, the isolation of *Salmonella* spp. and *Shigella* spp. in slightly lower proportions aligns with documented gastrointestinal disease burdens in SSA [48,49], where these bacteria, though not as prevalent as *E. coli,* contribute to high morbidity rates, especially in vulnerable populations such as the immunocompromised and children under the age of 5 years [4,25].

We observed that the common pathogens studied herein exhibited concerning resistance patterns to widely prescribed antibiotics in the region, which hinted limited effectiveness of antimicrobial therapy [50,51]. The observed high resistance to penicillins among *E. coli* and *Klebsiella* spp.*,* was consistent with reports made by earlier *s*tudies across SSA which linked high AMR to unregulated antibiotic use [4,52,53]. Our findings align with the World Health Organization’s Access, Watch, and Reserve (AWaRe) classification of antibiotics, which promotes prioritizing Access antibiotics, cautiously using Watch antibiotics, and reserving critical treatments as last-resort options [54]. In our study, high resistance was seen with piperacillin-tazobactam, which is a watch-category antibiotic, a finding much higher than in the current literature [55,56]. In the Reserve group, imipenem resistance (*E. coli* – 20.9%, *Klebsiella* spp. - 31.3%) was particularly concerning, as resistance to reserve antibiotics limits available options for treatment of multidrug-resistant (MDR) organisms [55,57], a trend that corroborates with similar studies in urban hospital settings [12]. In a similar study from Zambia that analyzed 34,672 isolates (2015–2020), susceptibility to imipenem was reported to be 46.2% and 50% among *E. coli* and *Klebsiella* spp. respectively [58].

Prioritizing access to antibiotics in regions where they remain effective may help control resistance rates in Watch and Reserve antibiotics, thereby extending their efficacy [54]. Given the high resistance observed to piperacillin-tazobactam and ampicillin, there is a need to avoid their usage for first-line treatments and develop strategic measures [59,60]. Empirical treatments in the region often depend on generalized data, which may not reflect local resistance patterns, increasing the risk of therapeutic failures in hospitals with diverse patient populations [61]. Updating treatment protocols in this region to emphasize susceptibility-based treatments could significantly impact the AMR trajectory. Such efforts require collaboration across sectors to ensure tailored interventions are sustainable and aligned with the One Health approach [62]. Careful monitoring of legacy antibiotics in both human and animal contexts is essential to prevent future resistance and preserve their usability [63,64]. Thus, these findings point to a need for strict regulatory frameworks for antibiotic use and integrated surveillance systems spanning clinical, animal, and environmental domains to manage resistance trends [61,65].

One major finding from our study was that chloramphenicol and third-generation cephalosporins like ceftazidime showed relatively higher susceptibility compared to other antibiotics; which could support their preference for patients with similar isolates. The fact that chloramphenicol, which inhibits protein synthesis via the 50S ribosomal subunit, retains efficacy may be attributed to the recent decline in its use that has caused resurgence in susceptibility [66,67]. A recent research work, the URbanZoo project [68], sampled *Escherichia coli* from humans, livestock and peri-domestic wildlife in 99 households across Nairobi in 2015–2016, revealing a significant transmission of AMR genes between humans and animals, corroborated by our study. Our study set in a rural and semi-urban setting displayed similar resistance patterns to penicillins, trimethoprim and sulfonamides. However, it was interesting to note that they detected a very low resistance level of 3% to chloramphenicol in livestock as compared to our 11% in animal feces and 72% in environmental samples. The high chloramphenicol resistance detected in environmental samples, even when chloramphenicol has been prohibited in food‐producing animals by the Kenyan government since 2010 [69], suggest that the environment acts as a long-term reservoir with the lingering circulation of resistance genes, and an impact on it needs longer [66,67]. Third-and fourth-generation cephalosporins like ceftazidime, ceftriaxone, cefotaxime, and cefepime target bacterial cell wall synthesis, and their slightly higher sensitivity rates may be related to their restricted access and relatively higher cost in SSA compared to other parts of the world [70]. However, rising resistance rates in Kenya and neighbouring countries, as reported in several studies [4], emphasize the need for prudent application to preserve their utility [12,19,71].

The high proportion of resistant *E. coli, Salmonella* spp.*,* and *Klebsiella* spp. strains across human, animal, water, and soil samples in our study adds to the evidence that animals and the environment act as reservoirs for ARGs, facilitating their transfer to humans through interconnected ecological pathways, for example, farmers working in direct contact with the environment and animals, complicating public health efforts [72,69]. These findings align with the One Health perspective, showing how antibiotic use in healthcare and agriculture shapes resistance in environmental bacteria, and underscore the role of selective pressures and horizontal gene transfer. Molecular analyses of the samples reported herein through whole-genome sequencing (WGS) will help to confirm potential relationships and transmission pathways. A similar study in China analyzed 592 samples across human, food, and environmental sectors using WGS, identified 40 ARG types and 743 ARG subtypes, and observed extensive ARG flow among One Health sectors [73]. Detailed comparison of resistance genes across human, animal, and environmental samples using sequencing results is planned for our study.

The prevalences of the ARGs isolated in this study agree with rates reported in other recent studies. Edwards et al. [29] reported high colonization by ESBL (89%) and carbapenemase (62.4%) producers in Kenyan and Nigerian neonatal units, with *CTX-M* and *NDM* being the most common genes. A meta-analysis of African water-plant-food interfaces found widespread ESBL-producing *Enterobacterales*, with *bla*CTX-M as the predominant genetic determinant [74]. Similarly, a review of East, Central, and Southern Africa reported a high prevalence of ESBL-producing *Enterobacterales*, particularly carrying *blaCTX-M-15* [75,76 ]. Whereas some studies [77,78] reported *blaOXA-181* as the most common gene in carbapenem-resistant *Enterobacterales* across human, animal, and environmental samples, our study noted *blaOXA*-48 as the most common one. This might be attributed to the general commonality of the genes reported herein in the locality [4,19,71]. Looking at the relative prevalences of ARGs in a One Health context supports this study’s assumption that agricultural antibiotic use/misuse and environmental contamination play a role in amplifying the spread of AMR.

It is alarming to note that all the collected drinking water samples were contaminated with *Enterobacterales* pathogens, interestingly with the highest resistance rate detected in the study against piperacillin-tazobactam in *E. coli*. This highlights the risk of waterborne AMR and its contribution towards community vulnerability to burden of infectious diseases and AMR [44,47,74]. Our results reiterate the dire need for improving drinking water conditions in Kenya, which was estimated to have 37% of good ambient water quality in its 2023 evaluation of the 6^th^ UN sustainable development goal for 2030 [79].

An important point of discussion triggered by our study finding is the disparities in the vigorous implementation of policy action plans and lack of availability of AMR One health data in Kenya and many other African countries. Recent literature [76,80,81] have portrayed the commendable progress made in the roll-out of the national action plan including policy changes, hospital and lab-based AMR surveillance setups, antibiotic stewardship efforts as well as digitalised data repositories and consolidation. They also throw light on the difficulties faced on ground including diagnostic deficiencies, decentralised healthcare and funding gaps. These highlight just the tip of the iceberg and demand immediate focus.

Gaps in health literacy were evidenced by the presence of unprescribed antibiotic use, a problem that has been highlighted in several studies across SSA [52,82]. Despite this, it is worth noting that over-the-counter antibiotics are common in the study area and most regions in Africa; therefore, better legislative measures and sensitization need to be put in place to reduce such cases, as they have significantly contributed to the emergence and re-emergence of AMR [13,52,53,82]. Most self-medicating individuals do not complete dosages and sometimes dispose of the remnants of the antibiotics into the environment, which will end up influencing environmental bacterial isolates towards developing resistance because of the selective pressure they impart on them [5,12,71].

Our study had some limitations. For instance, multivariate and subgroup analyses for factors related to susceptibility patterns could not be conducted owing to limited sample size and high prevalence rates. The seasonality of GI infections could have been missed as the study was not conducted throughout the year. Also, the small sample sizes limited the determination of a conclusive geographical distribution of the resistances across the region.

## 5. Conclusion

Our study revealed shared resistance patterns across human, animal, and environmental samples, highlighting interconnected AMR pathways. These findings underscore the urgent need for individually tailored AMR strategies in the SSA that address both clinical and environmental challenges. Localized AMR data, particularly in referral facilities, are critical for adapting treatment protocols to regional resistance patterns and reducing therapeutic failures. Comprehensive surveillance integrating human, animal, and environmental data is essential for targeted AMR control strategies. A One Health approach to AMR surveillance can generate localized, real-time data to inform treatment protocols, enabling healthcare providers to select effective antibiotics and mitigate the spread of resistant strains. Strengthened laboratory capacities to support laboratory data-guided prescriptions and targeted stewardship programs are critical. Regulatory measures and public health initiatives, including community education and training of healthcare workers, are essential to curb antibiotic misuse across sectors. These integrated efforts are vital to preserve antibiotic efficacy, improve patient outcomes, and address AMR holistically in Kenya and across SSA. By bridging gaps in diagnostics, surveillance, and stewardship, this pilot study provides a framework for addressing AMR effectively within a One Health paradigm. 

## Supporting information

S1 File Technical procedures and STROBE guidelines. (DOCX)

S2 File Consent forms used in the study. (DOCX)
