# Supplementary material for: One health approach unravels worrying antimicrobial resistance patterns: A cross-sectional study in Kisii, Kenya
Source: PLoS One. 2025 Sep 3;20(9):e0331389. doi: 10.1371/journal.pone.0331389 (PMC12407440; doi:10.1371/journal.pone.0331389)
Supplement: S1 File — (DOCX) [file pone.0331389.s001.docx]

**S1 File**

**Supplementary Material For**

**One health approach unravels worrying antimicrobial resistance patterns: a cross-sectional study in Kisii, Kenya.**

Briton M. Kavulavu, Eric O. Omwenga, Oscar Asanya Nyangiri, Andrew K. Nyerere, Rael J. Too, Elizabeth J. Matey, Siri Göpel, Wycliffe Mogoa, Thorben Schilling, Ludwig E. Hoelzle, Beryl Primrose Gladstone

**Table of Contents**

[Standard Laboratory Procedures 2](#_Toc198033439)

[A. Specimen Collection 2](#_Toc198033440)

[1. Human Stool Sample Collection 2](#_Toc198033441)

[2. Animal Stool Sample Collection 2](#_Toc198033442)

[3. Drinking Water Sample Collection 2](#_Toc198033443)

[4. Soil Sample Collection 2](#_Toc198033444)

[B. Culture and Isolation 3](#_Toc198033445)

[1. Human Stool Samples 3](#_Toc198033446)

[2. Animal Stool Samples 3](#_Toc198033447)

[3. Drinking Water Samples 3](#_Toc198033448)

[4. Soil Samples 3](#_Toc198033449)

[5. Identification of Colonies 3](#_Toc198033450)

[C. Antimicrobial Susceptibility Testing (AST) 4](#_Toc198033451)

[D. Molecular Characterization of ESBL and Carbapenemase-Producing Enterobacterales 5](#_Toc198033452)

[STROBE Checklist 7](#_Toc198033453)

# Standard Laboratory Procedures

## A. Specimen Collection (Ahmed, 2016; Bassetti et al., 2013; Horne et al., 2024; Jacob et al., 2020; Parija, 2023; Rawat et al., n.d.; Tambi et al., 2023; Watti et al., n.d.; Worku et al., 2022)

### 1. Human Stool Sample Collection

***1.1 Materials Required***

- Sterile specimen container
- Disposable gloves
- Transport bags

***1.2 Procedure***

1. Provide the patient with a sterile container for stool collection.
2. Instruct the patient to collect a fresh stool sample in the provided container. Ensure the sample is free from urine or water contamination.
3. Label the specimen with the study ID, collection date, and time.
4. Transport the stool sample to the laboratory within 1 hour of collection.

***1.3 Handling and Transport***

- Place the sample in a transport bag to prevent contamination during transport.
- Transport the sample to the laboratory immediately within 1 hour.

### 2. Animal Stool Sample Collection

***2.1 Materials Required***

- Sterile specimen containers
- Transport cooler with ice packs (for maintaining 4°C)
- Disposable gloves

***2.2 Procedure***

1. Collect 5g of less than 12-hour-old animal stool using sterile equipment (e.g., scoops or spatulas) from domestic animals within the patient’s household.
2. Place the collected stool in a sterile container immediately.
3. Label the specimen with the study ID, collection date, and time.
4. Transport the sample to the laboratory at 4°C within 2 hours of collection.

***2.3 Handling and Transport***

- Place the sample in a transport cooler with ice packs to maintain the required temperature (4°C).
- Transport the sample to the laboratory within 2 hours.

### 3. Drinking Water Sample Collection

***3.1 Materials Required***

- Sterile 250 mL water collection bottles
- Gloves
- Transport cooler with ice packs

***3.2 Procedure***

1. Collect 250 mL of water aseptically from a water source such as pots, jerricans, or dispensers where the patient drinks water.
2. Label the water sample with the study ID, collection date, and time.
3. Transport the sample to the laboratory within 2 hours at 4°C.

***3.3 Handling and Transport***

- Store the sample in a cooler with ice packs to maintain the temperature at 4°C during transport.
- Ensure the sample is transported to the laboratory within 2 hours.

### 4. Soil Sample Collection

***4.1 Materials Required***

- Sterile collection containers
- Shovel or sterile spatula
- Gloves

***4.2 Procedure***

1. Collect 5g of soil from the upper 5 cm of the soil surface in frequently contacted areas near the patient’s household.
2. Place the soil sample in a sterile container to avoid contamination.
3. Label the soil sample with the study ID, collection date, and time.
4. Transport the sample to the laboratory at room temperature within 2 hours.

**4.3 Handling and Transport**

- Ensure the sample is placed in a labeled, leak-proof container.
- Transport the sample to the laboratory at room temperature within 2 hours.

## B. Culture and Isolation (Ahmed, 2016; Bassetti et al., 2013; Horne et al., 2024; Jacob et al., 2020; Parija, 2023; Rawat et al., n.d.; Tambi et al., 2023; Watti et al., n.d.; Worku et al., 2022)

### 1. Human Stool Samples

1. Aliquot the stool sample into two portions:
   - Portion 1: Directly culture on MacConkey (MAC) Agar and incubate at 37°C for 18–24 hours.
   - Portion 2: Enrich in Selenite F broth at 37°C for 6-8 hours, then culture on Salmonella-Shigella (SS) Agar and incubate at 37°C for 18–24 hours.

### 2. Animal Stool Samples

1. Homogenize 1g of animal stool in sterile saline.
2. Culture on MAC Agar and incubate at 37°C for 18–24 hours.
3. Enrich the second aliquot in Selenite F broth at 37°C for 6-8 hours, then culture on SS Agar and incubate at 37°C for 18–24 hours.

### 3. Drinking Water Samples

1. Filter 100 mL of drinking water through a 0.45 µm membrane filter.
2. Culture the filter on MAC Agar and incubate at 37°C for 18–24 hours.
3. Enrich a second aliquot in buffered peptone water at 37°C for 6-8 hours, then culture on SS Agar at 37°C for 18–24 hours.

### 4. Soil Samples

1. Suspend 1g of soil in sterile saline, vortex, and allow it to settle.
2. Plate an aliquot of the supernatant on MAC Agar and incubate at 37°C for 18–24 hours.

### 5. Identification of Colonies

1. Describe colonies based on their morphological characteristics.
2. Identify the colonies by Gram staining and standard biochemical tests:
   - Triple Sugar Iron (TSI)
   - Motility
   - Indole
   - Citrate utilization
   - Urease tests.

**Table 1: Biochemical Reaction Patterns.**

| **Test** | | **Isolate reaction Pattern [n (%)]** | | | | ***E. coli* (ATCC 25922)** | ***K. pneumoniae* (ATCC 700603)** |
| --- | --- | --- | --- | --- | --- | --- | --- |
| **MacConkey Agar** | | LF | NLF | NLF | LF+ mucoid | LF | LF+ mucoid |
| **SS Agar** | | Pink | Colorless + black | Colorless | Pink | Pink | Pink |
| **Gram Staining** | | GNR | GNR | GNR | GNR | GNR | GNR |
| **TSI Agar** | **Slant** | A | K | K | A | A | A |
|  | **Butt** | A | A | A | A | A | A |
|  | **H₂S** | - | + | - | - | - | - |
| **Indole** | | + | - | - | - | + | - |
| **Citrate** | | - | + | - | + | - | + |
| **Urease** | | - | - | - | + | - | + |
| **Motility** | | + | + | - | - | + | - |
| **Probable**  **Pathogen** | | *Escherichia coli* | *Salmonella spp.* | *Shigella spp.* | *Klebsiella spp.* | ___ | ___ |
| LF: Lactose Fermenter; NLF: Non-Lactose Fermenter; GNR: Gram Negative Rods; A: Acidic (Yellow); K: Alkaline (Red); H₂S: Hydrogen Sulfide Production; +: Positive; -: Negative. | | | | | | | |

## C. Antimicrobial Susceptibility Testing (AST)

- **Preparation of Bacterial Suspensions**:
  - Select 3-5 isolated colonies from a fresh culture.
  - Suspend colonies in 4-5 mL of normal saline.
  - Adjust the turbidity manually to the 0.5 McFarland standard using a visual comparison.
- **Inoculation of Mueller-Hinton Agar**:
  - Dip a sterile cotton swab into the bacterial suspension.
  - Remove excess liquid by pressing the swab against the side of the tube.
  - Spread the suspension evenly onto Mueller-Hinton Agar plates using the lawn culture technique.
- **Antibiotic Testing**:
  - Antibiotics were selected based on CLSI 2024 guidelines (CLSI, 2024) and placed on the inoculated agar plates using forceps.
  - The selected antibiotics were as follows:
    - Amoxicillin-clavulanate (AMC, 20/10 µg)
    - Ampicillin (AMP, 10 µg)
    - Trimethoprim-sulfamethoxazole (SXT, 1.25/23.75 µg)
    - Imipenem (IPM, 10 µg)
    - Cefotaxime (CTX, 30 µg)
    - Ceftazidime (CAZ, 30 µg)
    - Amikacin (AK, 30 µg)
    - Ceftriaxone (CRO, 30 µg)
    - Piperacillin-tazobactam (TZP, 100/10 µg)
    - Kanamycin (KAN, 30 µg)
    - Cefepime (FEP, 30 µg)
    - Chloramphenicol (CHL, 30 µg)
    - Ciprofloxacin (CIP, 5 µg)
  - Incubate the plates at 35°C for 16-18 hours.
- **Measurement of Inhibition Zones**:
  - After incubation, measure the diameters of inhibition zones manually.
  - Classify isolates as susceptible, intermediate, or resistant based on CLSI 2024 guidelines (42).
- **Detection of Extended-Spectrum Beta-Lactamases (ESBLs)**:
  - Use the double-disk synergy method with cefotaxime (30 µg), ceftazidime (30 µg), and their combinations with clavulanate (30/10 µg) (Silago et al., 2021).
    - Positive: A ≥5 mm increase in the inhibition zone around the clavulanate combination disk compared to individual antibiotic disks indicates ESBL production.
    - Negative: No significant increase in inhibition zone around the clavulanate disk.

## D. Molecular Characterization of ESBL and Carbapenemase-Producing Enterobacterales

- **DNA Extraction**:
  - Use Invitrogen™ PureLink™ Genomic DNA Mini Kit.
  - Lyse cells with Proteinase K, remove RNA with RNase A, and bind DNA with ethanol on a spin column.
  - Elute DNA with PureLink™ Genomic Elution Buffer and store at -20°C.
- **PCR**:
  - Prepare a 25 μL reaction:
    - 12.5 μL Thermo Scientific™ DreamTaq PCR Master Mix (2X)
    - 1 μL each of forward and reverse primers
    - 2 μL DNA template
    - 8.5 μL nuclease-free water
  - Amplification cycle:
    - Initial denaturation: 94°C for 5 minutes
    - 30 cycles of:
      - Denaturation: 95°C for 30 seconds
      - Annealing: temperature based on primer details (see Table 2) for 30 seconds
      - Extension: 72°C for 30 seconds
    - Final extension: 72°C for 10 minutes.
- **Electrophoresis**:
  - Separate PCR products on a 1% agarose gel with SYBR™ Safe DNA Gel Stain.
  - Use a 100–1,000 bp GeneRuler DNA Ladder to determine the product size.
  - Run gel at appropriate voltage for 30-60 minutes and visualize the bands under UV light to confirm the presence of the targeted genes.

**Table 2: List of primer sequences used for screening ESBL and carbapenemase-encoding genes.**

| **Gene name** | **Primer sequence** | **Annealing Temp** | **Fragment size(bp)** | **Reference** |
| --- | --- | --- | --- | --- |
| blaTEM | F: AAACGCTGGTGAAAGTA  R: AGCGATCTGTCTAT | 46^o^C | 822 | (Mohamed et al., 2020) |
| blaSHV | F: ATGCGTTATATTCGCCTGTG  R: TGCTTTGTTATTCGGGCCAA | 53^o^C | 753 |  |
| blaCTX-M-1 | F: GGT TAA AAA ATC ACT GCG TC  R: TTG GTG ACG ATT TTA GCC GC | 55^o^C | 850 |  |
| blaCTX-M - 9 | F: ATG GTG ACA AAG AGA GTG CA  R: CCC TTC GGC GAT GAT TCT C | 53^o^C | 850 |  |
| blaCTX-M − 8 | F: TCGCGTTAAGCGGATGATGC  R: AACCCACGATGTGGGTAG | 57^o^C | 666 |  |
| blaCTX-M-15 | F: GTGATACCACTTCACCTC  R: AGTAAGTGACCAGAATCAG | 53^o^C | 255 | (Khalifa et al., 2021) |
| blaVIM | F: GATGGTGTTTGGTCGCATA  R: CGAATGCGCAGCACCAG | 53^o^C | 390 | (Poirel et al., 2011) |
| blaNDM | F: GGTTTGGCGATCTGGTTTTC  R: CGGAATGGCTCATCACGATC | 53^o^C | 621 |  |
| blaKPC | F: CGTCTAGTTCTGCTGTCTTG  R: CTTGTCATCCTTGTTAGGCG | 53^o^C | 798 |  |
| bla_IMP_ | F: GGAATAGAGTGGCTTAAYTCTC  R: GGTTTAAYAAAACAACCACC | 52^o^C | 232 |  |
| blaOXA-48 | F: GCGTGGTTAAGGATGAACAC  R: CATCAAGTTCAACCCAACCG | 53^o^C | 438 |  |

# STROBE Checklist

|  | Item No. | Recommendation | Page  No. | | Relevant text from the manuscript |
| --- | --- | --- | --- | --- | --- |
| **Title and abstract** | 1 | (*a*) Indicate the study’s design with a commonly used term in the title or the abstract | 1 | **Line 1** | |
|  |  | (*b*) Provide in the abstract an informative and balanced summary of what was done and what was found | 2 | **Lines 23 to 59** | |
| Introduction | | | |  | |
| Background/rationale | 2 | Explain the scientific background and rationale for the investigation being reported | 3 | Lines 61 to 104 | |
| Objectives | 3 | State specific objectives, including any prespecified hypotheses | 4 | Lines 105 to 112 | |
| Methods | | | |  | |
| Study design | 4 | Present key elements of study design early in the paper | 5 | Lines 115 to 117 | |
| Setting | 5 | Describe the setting, locations, and relevant dates, including periods of recruitment, exposure, follow-up, and data collection | 5 | Lines 117 to 121 | |
| Participants | 6 | (*a*) *Cohort study*—Give the eligibility criteria, and the sources and methods of selection of participants. Describe methods of follow-up  *Case-control study*—Give the eligibility criteria, and the sources and methods of case ascertainment and control selection. Give the rationale for the choice of cases and controls  *Cross-sectional study*—Give the eligibility criteria, and the sources and methods of selection of participants | 5 | | Lines 123 to 125 |
|  |  | (*b*) *Cohort study*—For matched studies, give matching criteria and number of exposed and unexposed  *Case-control study*—For matched studies, give matching criteria and the number of controls per case | n/a | |  |
| Variables | 7 | Clearly define all outcomes, exposures, predictors, potential confounders, and effect modifiers. Give diagnostic criteria, if applicable | 6 | | *Lines 131 to 158* |
| Data sources/ measurement | 8* | For each variable of interest, give sources of data and details of methods of assessment (measurement). Describe comparability of assessment methods if there is more than one group | 6 | | *Lines 131 to 158* |
| Bias | 9 | Describe any efforts to address potential sources of bias | n/a | |  |
| Study size | 10 | Explain how the study size was arrived at | 5 | | Lines 115 to 116 |

Continued on next page

| Quantitative variables | 11 | Explain how quantitative variables were handled in the analyses. If applicable, describe which groupings were chosen and why | 7 | Lines 164 to 165 |
| --- | --- | --- | --- | --- |
| Statistical methods | 12 | (*a*) Describe all statistical methods, including those used to control for confounding | 7 | Lines 166 to 169 |
|  |  | (*b*) Describe any methods used to examine subgroups and interactions | 7 | Lines 169 and 169 |
|  |  | (*c*) Explain how missing data were addressed | n/a |  |
|  |  | (*d*) *Cohort study*—If applicable, explain how loss to follow-up was addressed  *Case-control study*—If applicable, explain how matching of cases and controls was addressed  *Cross-sectional study*—If applicable, describe analytical methods taking account of sampling strategy | 7 | Lines 165 to 169 |
|  |  | (*e*) Describe any sensitivity analyses | n/a |  |
| Results | | | | |
| Participants | 13* | (a) Report numbers of individuals at each stage of study—eg numbers potentially eligible, examined for eligibility, confirmed eligible, included in the study, completing follow-up, and analysed | 8 | Line 182 |
|  |  | (b) Give reasons for non-participation at each stage | 7 | Line 177 |
|  |  | (c) Consider use of a flow diagram |  |  |
| Descriptive data | 14* | (a) Give characteristics of study participants (eg demographic, clinical, social) and information on exposures and potential confounders | 8 | Lines 182 to 193 |
|  |  | (b) Indicate number of participants with missing data for each variable of interest | n/a |  |
|  |  | (c) *Cohort study*—Summarise follow-up time (eg, average and total amount) |  |  |
| Outcome data | 15* | *Cohort study*—Report numbers of outcome events or summary measures over time |  |  |
|  |  | *Case-control study—*Report numbers in each exposure category, or summary measures of exposure |  |  |
|  |  | *Cross-sectional study—*Report numbers of outcome events or summary measures | *9-25* | *Lines 196-334* |
| Main results | 16 | (*a*) Give unadjusted estimates and, if applicable, confounder-adjusted estimates and their precision (eg, 95% confidence interval). Make clear which confounders were adjusted for and why they were included | n/a |  |
|  |  | (*b*) Report category boundaries when continuous variables were categorized | 8 | Line 194 |
|  |  | (*c*) If relevant, consider translating estimates of relative risk into absolute risk for a meaningful time period | n/a |  |

Continued on next page

| Other analyses | 17 | Report other analyses done—eg analyses of subgroups and interactions, and sensitivity analyses | n/a |  |
| --- | --- | --- | --- | --- |
| Discussion | | | | |
| Key results | 18 | Summarise key results with reference to study objectives | 25 | Lines 337 to 346 |
| Limitations | 19 | Discuss limitations of the study, taking into account sources of potential bias or imprecision. Discuss both direction and magnitude of any potential bias | 29 | Lines 436 to 440 |
| Interpretation | 20 | Give a cautious overall interpretation of results considering objectives, limitations, multiplicity of analyses, results from similar studies, and other relevant evidence | 26-30 | Lines 347 to 440 |
| Generalisability | 21 | Discuss the generalisability (external validity) of the study results | 30 | Lines 441 to 455 |
| Other information | |  | | |
| Funding | 22 | Give the source of funding and the role of the funders for the present study and, if applicable, for the original study on which the present article is based | 32 | Line 486 |

**Referneces**

Ahmed, S. (2016). *Culture, Isolation and Identification of Bacteria From Municipal Area of Soil*. https://doi.org/10.13140/RG.2.2.11306.03525

Bassetti, M., Merelli, M., Temperoni, C., & Astilean, A. (2013). New antibiotics for bad bugs: Where are we? *Annals of Clinical Microbiology and Antimicrobials*, *12*(1), 22. https://doi.org/10.1186/1476-0711-12-22

CLSI. (2024). *M100 Ed34 | Performance Standards for Antimicrobial Susceptibility Testing, 34th Edition*. Clinical Laboratory Standards Institute. https://clsi.org/standards/products/microbiology/documents/m100/

Horne, B., Badji, H., Bhuiyan, M. T. R., Romaina Cachique, L., Cornick, J., Hotwani, A., Juma, J., Ochieng, J. B., Abdou, M., Apondi, E., Atlas, H. E., Awuor, A. O., Baker, K. S., Ceesay, B. E., Charles, M., Cunliffe, N. A., Feutz, E., Galagan, S. R., Guindo, I., … Tennant, S. M. (2024). Microbiological Methods Used in the Enterics for Global Health Shigella Surveillance Study. *Open Forum Infectious Diseases*, *11*(Supplement_1), S25–S33. https://doi.org/10.1093/ofid/ofad576

Jacob, M. E., Keelara, S., Aidara-Kane, A., Matheu Alvarez, J. R., & Fedorka-Cray, P. J. (2020). Optimizing a Screening Protocol for Potential Extended-Spectrum β-Lactamase Escherichia coli on MacConkey Agar for Use in a Global Surveillance Program. *Journal of Clinical Microbiology*, *58*(9), 10.1128/jcm.01039-19. https://doi.org/10.1128/jcm.01039-19

Khalifa, S. M., Abd El-Aziz, A. M., Hassan, R., & Abdelmegeed, E. S. (2021). β-lactam resistance associated with β-lactamase production and porin alteration in clinical isolates of E. coli and K. pneumoniae. *PloS One*, *16*(5), e0251594. https://doi.org/10.1371/journal.pone.0251594

Mohamed, E. S., Khairy, R. M. M., & Abdelrahim, S. S. (2020). Prevalence and molecular characteristics of ESBL and AmpC β -lactamase producing Enterobacteriaceae strains isolated from UTIs in Egypt. *Antimicrobial Resistance & Infection Control*, *9*(1), 198. https://doi.org/10.1186/s13756-020-00856-w

Parija, S. C. (2023). Salmonella and Shigella. In S. C. Parija (Ed.), *Textbook of Microbiology and Immunology* (pp. 517–540). Springer Nature. https://doi.org/10.1007/978-981-19-3315-8_36

Poirel, L., Walsh, T. R., Cuvillier, V., & Nordmann, P. (2011). Multiplex PCR for detection of acquired carbapenemase genes. *Diagnostic Microbiology and Infectious Disease*, *70*(1), 119–123. https://doi.org/10.1016/j.diagmicrobio.2010.12.002

Rawat, N. S., Lathwal, S. S., Panchbhai, G. J., Jha, A. K., & Gupta, S. K. (n.d.). *Physical and microbial characteristics of fresh urine and dung of heifer and lactating Sahiwal cow*.

Silago, V., Kovacs, D., Samson, H., Seni, J., Matthews, L., Oravcová, K., Lupindu, A. M., Hoza, A. S., & Mshana, S. E. (2021). Existence of Multiple ESBL Genes among Phenotypically Confirmed ESBL Producing Klebsiella pneumoniae and Escherichia coli Concurrently Isolated from Clinical, Colonization and Contamination Samples from Neonatal Units at Bugando Medical Center, Mwanza, Tanzania. *Antibiotics*, *10*(5), Article 5. https://doi.org/10.3390/antibiotics10050476

Tambi, A., Brighu, U., & Gupta, A. B. (2023). *(PDF) Methods for detection and enumeration of coliforms in drinking water: A review*. *23*(1). https://www.researchgate.net/publication/374248877_Methods_for_detection_and_enumeration_of_coliforms_in_drinking_water_a_review

Watti, V. M., Wafula, N., Fungo, R., & Mahungu, S. (n.d.). Prevalence of waterborne pathogens in drinking water from different sources: A trans-boundary study in the Nile Basin. *World Water Policy*, *n/a*(n/a). https://doi.org/10.1002/wwp2.12240

Worku, M., Getie, M., Moges, F., & Mehari, A. G. (2022). Extended-Spectrum Beta-Lactamase- and Carbapenemase-Producing Enterobacteriaceae Family of Bacteria from Diarrheal Stool Samples in Northwest Ethiopia. *Interdisciplinary Perspectives on Infectious Diseases*, *2022*, 7905350. https://doi.org/10.1155/2022/7905350
