# Supplementary material for: One health approach unravels worrying antimicrobial resistance patterns: A cross-sectional study in Kisii, Kenya
Source: PLoS One. 2025 Sep 3;20(9):e0331389. doi: 10.1371/journal.pone.0331389 (PMC12407440; doi:10.1371/journal.pone.0331389)
Supplement: S2 File — (DOCX) [file pone.0331389.s002.docx]

**S2 File**

**Supplementary Material For**

**One health approach unravels worrying antimicrobial resistance patterns: a cross-sectional study in Kisii, Kenya.**

Briton M. Kavulavu, Eric O. Omwenga, Oscar Asanya Nyangiri, Andrew K. Nyerere, Rael J. Too, Elizabeth J. Matey, Siri Göpel, Wycliffe Mogoa, Thorben Schilling, Ludwig E. Hoelzle, Beryl Primrose Gladstone

**Table of Contents**

[Adult Consent Forms 2](#_Toc198037479)

[English Version 2](#_Toc198037480)

[Swahili Version 6](#_Toc198037481)

[Assent form 12 to 17 Years 11](#_Toc198037482)

[English Version 11](#_Toc198037483)

[Swahili Version 14](#_Toc198037484)

[Parental Permission Form 17](#_Toc198037485)

[English Version 17](#_Toc198037486)

[Swahili Version 21](#_Toc198037487)

## ****Adult Consent Forms****

### English Version

**Patient Information Sheet**

Dear Sir/Madam,

You have been invited to participate in this research study. Before you decide to participate, please read this information sheet carefully.

**1) Who is organizing and financing this study?**

This study is organized by the Kisii University school of Medicine and Kisii University school of Health Sciences and has been approved by the Moi University Research ethics committee.

It is part of a pilot study "Natural history and transmission dynamics of antimicrobial resistance in an East African set-up” funded by the Universities Hohenheim and Tübingen Project in Germany.

**2) What is KONSERVE-AB?**

KONSERVE-AB is a collaborative project that aims to better understand the spread and persistence of antibiotic resistant bacteria in the community and more specifically within the humans, animals and the environment. The analysis methods at the molecular level will provide new insights into the understanding of the transmission and persistence of antibiotic resistant bacteria within a community. The results will help prioritize interventions to address the problem of antibiotic resistance.

**3) What is the purpose of this study?**

We all carry a large number of non-harmful bacteria that live in our gut, also known as gut flora. When taking antibiotics, these bacteria can develop resistance to antibiotics called (Extended-spectrum beta-lactamase-producing Enterobacteriaceae) ESBL-PE. In simple terms, the bacteria produce substances to break down antibiotics and therefore cannot be killed by these antibiotics. The main reservoir for patients colonized by these ESBL-PE bacteria is the digestive tract. It could be transmitted through animals as well. The continued spread of antibiotic-resistant bacteria is one of the most important problems in antimicrobial resistance. It is a worldwide threat to public health, not only in health care facilities but also in the community.

The purpose of the KONSERVE-AB study is to assess whether bacterial resistance in individuals is transmitted from or to animals or environment in their households. It aims to determine the amount of these infections and risk factors.

It is planned to include 200 patients in Kisii from May to September 2021. For resistant patients we will collect data from their household environment including animal droppings, soil and water. This is an observational study, and no experimental treatment is administered.

**4) Why have I been chosen?**

You were chosen for one of two reasons:

You are in the hospital with fever and diarrhoea and you are suspected to have enterobacterioceae bacterial infection in your gut.

**5) Who cannot participate?**

You cannot participate if you live more than 25km from Kisii. In addition, those who refuse to give consent or have been subjected to antibiotic usage will be excluded.

**6) Can I be excluded from the study after I give my consent?**

The study investigators can decide at any time to terminate your participation if it is in your medical interest to do so, without having to obtain your prior consent. You will be informed of the reason for the termination.

**7) Do I have to take part in this study and what are my rights?**

Your participation in this study is completely voluntary. Not taking part in the study will not affect your future medical care. The same principle applies if you revoke your initial consent after starting the study. You can therefore withdraw your participation at any time without having to justify your decision. In the event of revocation, the data collected up to that point will continue to be used.

**8) What should I do if I participate in this study?**

Participants will have the tests described below.

Group 1: If you agree to take part in this study, the study investigator will ask you to sign a consent form to confirm that you understand the study and agree to participate. Your participation in the study will be recorded in your computerized care record. You will not receive any experimental treatment. You will be asked questions about your illness, medical history, contact with animals and dietary preferences. You will be asked to provide stool sample. If your sample tests positive for the resistant Enterobacteriaceae bacteria, environmental samples such as water sample, soil, animal stool sample if any will be collected from your household.

**9) What are my obligations in the study?**

As a study participant, you are required to follow and respect the study design. During the house visit, you will be requested to provide environmental samples.

**10) What microbiological samples will be taken? Will these tests cause any discomfort?**

In order to search for the presence of resistant bacteria, a stool sample is necessary. This sampling does not cause any physical discomfort. If your sample tests positive for the resistant Enterobacteriaceae bacteria, environmental samples such as water sample, soil, animal stool sample if any will be collected from your household.

You will receive a special device with instructions on how to collect a stool sample by yourself in a simple and hygienic way.

**11) What happens to the samples once they are collected?**

A study investigator will collect the samples from you. They will be centralized, analyzed at the Kisii University school of health sciences laboratory and used for research purposes. They will be treated in a strictly confidential and anonymous manner; personal identifiers will be replaced by a code. The only people who have access to this code are the principal investigator of the study, Dr Wycliffe Mogoa, and his collaborators who are in charge of the study. They will then be kept for 10 years. You have the right to see the data and to have the samples destroyed. Any resistant bacteria found in any of the samples will be isolated and compared with each other in the German collaborative University, the Hohenheim University.

**12) What are the benefits and drawbacks of the study?**

Participation in the study does not create any direct benefit for you apart from the knowledge of the antibiotic resistance in your intestinal flora and the possible transmission within your family. The results of the test will be within 5 to 8 days and will inform your doctor if the prescribed antibiotic is appropriate or whether there is need to change treatment. With your agreement, the results of the microbiological analysis will be communicated to you and your doctor. You have the option to visit the hospital to ask what it means for your treatment.

The main benefit is that you will help improve the understanding of antimicrobial resistance, its transmission and persistence. The results of this research may help to improve antibiotic treatment of infections with resistant bacteria in the future.

The main disadvantage of participation is the time required for sample collection, and the questionnaire.

**13) What will these results be used for?**

The results of this study will be shared with other researchers and physicians in Kenya and Europe. They will be used to improve antibiotic treatment and minimize the risk of antibiotic resistance.

**14) Will I be informed of new findings from the study?**

The doctor will inform you of the results of your samples. Once the study is completed and analyzed, you will also be informed of the results in writing.

**15) Is my participation confidential?**

Your personal and medical data collected during the study will be treated as confidential.

Only the investigators of this study, the representatives of the Ethics Committee of the Kisii University are authorized to examine the patients' medical records.

Analyses may be published in scientific journals but your name will not appear in these journals. All persons having access to your medical records will be bound by confidentiality and your identity will never be revealed.

Your attending physician will be informed of your participation in this study. If necessary, he or she will be able to provide us with information about your follow-up. However, your doctor will not see your answers to the questionnaire. Study records will be kept for 10 years. All information will be kept strictly confidential and microbiological samples will be processed exclusively by the laboratories participating in the study. No other tests will be performed on the microbiological samples other than those planned for this study. Your contact information will only be given to the local study investigators.

**16) Will there be any personal or health insurance costs associated with my participation in this study?**

There will be no additional cost to you for participating in this study. The stool testing for presence of resistant bacteria will be of no cost for you.

Thank you for taking the time to read this information sheet and for considering participating in this study. If you want to participate, your doctor will ask you to sign a consent form.

**Contact Information for questions about the study**

If you have any more questions, please feel free to contact the principal investigators of the study:

Kisii University

School of Health sciences

P. O. Box 408-40200

Along Kisii-Kilgoris road

Kisii

Dr Wycliffe Mogoa

Email: [som@kisiiuniversity.ac.ke](mailto:som@kisiiuniversity.ac.ke)

Dr Oscar Asanya Nyangiri

Email: [oasanya@gmail.com](mailto:oasanya@gmail.com)

Dr Eric Omwenga Omori

E-mail: [omorieric@kisiiuniversity.ac.ke](mailto:omorieric@kisiiuniversity.ac.ke)

Questions about your rights as a participant: You may contact the Institutional Ethics and Research Committee (MTRH//MU-IREC) 0787723677 or email irec@mtrh.go.ke or [irecoffice@gmail.com](mailto:irecoffice@gmail.com). The MTRH//MU-IREC is a group of people that review studies for safety and to protect the rights of participants.

**Informed Consent Form**

**Part I : Consent For Participation In The Study**

| **KISII Onehealth amr project**  **(Konserve-AB)** | |
| --- | --- |
| **Location of the study: Kisii University School of Medicine,**  **Kisii, Kenya** | |
| **Investigator/ Co-investigator:** | |
| **Patient :**  Surname, Name | |
| **Date of birth:** \|___\|___\| \|___\|___\| \|__\|__\|___\|___\| | Sex : O male O female |

- I declare that I have been informed orally and in writing by the signing health worker about the above mentioned study.
- I confirm that I have read and understood the information to participants that was given to me (version 1. of 28.03.2021) and that I have had the opportunity to ask questions. I have received a copy of the information and consent form.
- I understand that my participation is voluntary and that I am free to withdraw from the study at any time, without giving any reason, without affecting my medical care or legal rights.
- I confirm that I have had sufficient time to make a decision regarding my participation in the study
- I have been informed of the obligation of the Kisii University to compensate me for any damage that may occur in the course of this study.
- I understand that certain parts of my medical file in relation to the study will be examined by researchers at the University Hospitals of Kisii or by regulatory authorities, while respecting confidentiality. I give permission to these individuals to have access to my records, including my contact information, and to be contacted by the investigators.
- I give my consent for the investigators to obtain medical information about me from my treating physician.
- I give my consent to participate in the above mentioned study.
- I agree to my stool being tested for resistance to antibiotics only
- I agree to my stool being tested for resistance to antibiotics and also a home visit to collect environmental samples

| Place, date | Signature of the patient |
| --- | --- |
| Place, date | Name and signature of the Principal Investigator / Co-investigator |

**If illiterate**

*A literate witness must sign (if possible, this person should be selected by the participant and should have no connection to the research team). Participants who are illiterate should include their thumb-print as well.*

I have witnessed the accurate reading of the consent form to the potential participant, and the individual has had the opportunity to ask questions. I confirm that the individual has given consent freely.

***Print name of witness_____________________ AND Thumb print of participant***

***Signature of witness ______________________***

**Date ________________________**

**Day/month/year**

**Part II. Informed Consent Form for Consent for Storage and Future Use of Unused Samples**

If the stool sample I have provided for this research project is unused or leftover when the project is completed (Tick **one** choice from each of the following boxes)

- I wish my stool sample to be destroyed immediately.
- I want my stool sample to be destroyed after ____ years.
- I give permission for my stool sample to be stored indefinitely

AND (if the sample is to be stored)

- I give permission for my stool sample to be stored and used in future research but only on the same subject as the current research project: KONSERVE-AB
- I give my permission for my stool sample to be stored and used in future research of any type which has been properly approved
- I give permission for my stool sample to be stored and used in future research except for research about __________________________

AND

- I want my identity to be removed from my stool sample.
- I want my identity to be kept with my stool sample.

I have read the information, or it has been read to me. I have had the opportunity to ask questions about it and my questions have been answered to my satisfaction. I consent voluntarily to have my samples stored in the manner and for the purpose indicated above.

**Print Name of Participant__________________**

**Signature of Participant ___________________**

**Date ___________________________**

**Day/month/year**

**If illiterate**

*A literate witness must sign (if possible, this person should be selected by the participant and should have no connection to the research team). Participants who are illiterate should include their thumb-print as well.*

I have witnessed the accurate reading of the consent form to the potential participant, and the individual has had the opportunity to ask questions. I confirm that the individual has given consent freely.

***Print name of witness_____________________ AND Thumb print of participant***

***Signature of witness ______________________***

**Date ________________________**

**Day/month/year**

**Statement by the researcher/person taking consent**

- I have accurately read out the information sheet to the potential participant, and to the best of my ability made sure that the participant understands that the sample will be stored for future use in accordance to the participant’s choice ticked above.
- I confirm that the participant was given an opportunity to ask questions about the nature and manner of storage of the samples, and all the questions asked by the participant have been answered correctly and to the best of my ability. I confirm that the individual has not been coerced into giving consent, and the consent has been given freely and voluntarily.

**A copy of this ICF has been provided to the participant.**

Print Name of Researcher/person taking the consent________________________

Signature of Researcher /person taking the consent__________________________

Date ___________________________

Day/month/year

### Swahili Version

**Habari kwa washiriki**

**Karatasi ya Habari ya Wagonjwa**

Bwana / Bi,

Umealikwa kushiriki katika utafiti huu. Kabla ya kuamua kushiriki, tafadhali soma karatasi hii ya habari kwa umakini.

1. **Ni nani wanaoendesha na kufadhili utafiti huu?**

Utafiti huu umeandaliwa na shule ya Tiba ya Chuo Kikuu cha Kisii na shule ya Chuo Kikuu cha Kisii ya Sayansi ya Afya na imeidhinishwa na kamati ya maadili ya Utafiti ya chou kikuu cha Moi. Ni sehemu ya utafiti wa majaribio "Historia ya asili na mienendo ya kuenea kwa viini sugu katika Afrika Mashariki" uliofadhiliwa na Mradi wa Vyuo Vikuu Hohenheim und Tübingen nchini Ujerumani.

1. **KONSERVE-AB ni nini?**

KONSERVE-AB ni mradi wa kushirikiana ambao unakusudia kuelewa vizuri kuenea na kuendelea kwa bakteria sugu ya antibiotic katika jamii na haswa ndani ya wanadamu, wanyama na mazingira. Mbinu ya uchambuzi wa DNA ya bakteria zitatoa ufahamu mpya juu ya uelewa wa usambazaji na kuendelea kukaa kwa bakteria zlizozo sugu kwa antibiotic ndani ya jamii. Matokeo yatasaidia kuweka kipaumbele hatua za kushughulikia shida ya upinzani wa antibiotic.

1. **Kusudi la utafiti huu ni nini?**

Sisi sote hubeba idadi kubwa ya bakteria wasio na madhara ambao hukaa ndani ya utumbo wetu, pia hujulikana kama mimea ya utumbo. Wakati tunakunywa dawa ya antibiotic, mimea asili ya tumbo na pia bakteria inayosababisha magonjwa hutoa madini yanayoharibu antibiotic ili ziwe sugu. Madini haya yanaitwa ESBL na yakiadhiri utumbo wale bakteia wanayoyatoa huitwa ESBL-PE. Kuenea kwa bakteria sugu ya dawa ni moja wapo ya shida muhimu katika upinzani wa antimicrobial.

Kuenea kwa bakteria sugu ya dawa ni moja wapo ya shida muhimu katika upinzani wa antimicrobial. Ni tishio ulimwenguni kwa afya ya umma, sio tu katika vituo vya huduma za afya lakini pia katika jamii.

Madhumuni ya utafiti wa KONSERVE-AB ni kutathmini ikiwa upinzani wa bakteria kwa watu binafsi hupitishwa kutoka au kwa wanyama au mazingira katika kaya zao. Inalenga kuamua kiwango cha maambukizo haya na sababu zinazoweka watu kwa hatari ya maambukizi haya. Imepangwa kujumuisha wagonjwa 200 huko Kisii kuanzia Mei hadi Septemba 2021.

Kwa wagonjwa sugu tutakusanya data kutoka kwa mazingira ya nyumba zao pamoja na kinyesi cha wanyama, udongo na maji. Huu ni utafiti wa uchunguzi, na hakuna matibabu ya majaribio yanayotekelezwa.

1. **Kwa nini nimechaguliwa?**

Ulichaguliwa kwa sababu moja wapo:

Uko hospitalini na homa na kuhara na unashukiwa kuwa na maambukizo ya bakteria ya enterobacteriaceae ndani ya utumbo wako.

1. **Nani hawezi kushiriki?**

Hauwezi kushiriki ikiwa unaishi mbali na Kisii.

1. **Je! Ninaweza kutengwa kwenye utafiti baada ya kutoa idhini yangu?**

Wachunguzi wa utafiti wanaweza kuamua wakati wowote kukomesha ushiriki wako ikiwa ni kwa masilahi yako ya matibabu kufanya hivyo, bila kupata idhini yako ya hapo awali. Utaarifiwa sababu ya kukomesha.

1. **Je! Lazima nishiriki katika utafiti huu na haki zangu ni zipi?**

Ushiriki wako katika utafiti huu ni wa hiari kabisa. Kutokushiriki katika utafiti hakuwezi kuathiri matibabu yako ya baadaye. Kanuni hiyo hiyo inatumika ikiwa utabadilisha idhini yako ya kwanza baada ya kuanza utafiti. Kwa hivyo unaweza kuondoa ushiriki wako wakati wowote bila kuhalalisha uamuzi wako. Katika tukio la kufutwa, data iliyokusanywa hadi wakati huo itaendelea kutumiwa.

1. **Nifanye nini ikiwa nitashiriki katika utafiti huu?**

Washiriki watakuwa na vipimo vilivyoelezewa hapa chini.

Kikundi cha 1: Ikiwa unakubali kushiriki katika utafiti huu, mchunguzi wa utafiti atakuuliza utia saini fomu ya idhini ili kuthibitisha kuwa unaelewa utafiti huo na unakubali kushiriki. Ushiriki wako katika utafiti utarekodiwa katika rekodi yako ya utunzaji wa kompyuta. Hautapokea matibabu yoyote ya majaribio. Utaulizwa maswali juu ya ugonjwa wako, historia ya matibabu, mawasiliano na wanyama na upendeleo wa lishe. Utaulizwa kutoa sampuli ya kinyesi. Ikiwa vipimo vyako vya sampuli vinafaa kwa bakteria sugu ya Enerobacteriaceae, sampuli za mazingira kama vile sampuli ya maji, mchanga, sampuli ya kinyesi cha wanyama ikiwa itakusanywa kutoka kwa kaya yako.

1. **Majukumu yangu katika utafiti ni nini?**

Kama mshiriki wa utafiti, unahitajika kufuata na kuheshimu muundo wa utafiti. Wakati wa ziara ya boma, utaombwa kutoa sampuli za mazingira.

1. **Ni sampuli gani za microbiolojia zitakazochukuliwa? Je! Vipimo hivi vitasababisha usumbufu wowote?**

Ili kutafuta uwepo wa bakteria sugu, sampuli ya kinyesi ni muhimu. Sampuli hii haisababishi usumbufu wowote wa mwili. Ikiwa vipimo vyako vya sampuli vitapatikana na bakteria sugu za Enterobacteriaceae, sampuli za mazingira kama vile sampuli ya maji, mchanga, sampuli ya kinyesi cha mifugu kama wapo itakusanywa kutoka kwa boma yako.

Utapokea kifaa maalum na maagizo ya jinsi ya kukusanya sampuli ya kinyesi na wewe mwenyewe kwa njia rahisi na ya usafi.

1. **Ni nini hufanyika kwa sampuli mara zinapokusanywa?**

Mchunguzi wa utafiti atakusanya sampuli kutoka kwako. Yatawekwa pamoja, kuchambuliwa katika shule ya Chuo Kikuu cha Kisii ya maabara ya sayansi ya afya na kutumika kwa madhumuni ya utafiti. Sampuli itashughulikiwa kwa njia ya siri na ambayo haitambulishi; yaani nambari itatumiwa badala ya vitambulisho vya kibinafsi.

Watu pekee ambao watajua nambari zitakazotumika ni mchunguzi mkuu wa utafiti huu, Dk Wycliffe Mogoa, na washirika wake ambao wanasimamia utafiti huu. Sampuli zitahifadhiwa kwa miaka 10. Una haki ya kuona data yako na kuitisa sampuli yako iharibiwe. Bakteria yoyote sugu inayopatikana katika sampuli yoyote itatengwa na ikilinganishwa na kila mmoja katika Chuo Kikuu cha Ushirika cha Ujerumani, Chuo Kikuu cha Hohenheim.

1. **Je! Kuna faida gani na mapungufu gani katika utafiti huu?**

Kushiriki katika utafiti italeta ufahamu wa upinzani wa antibiotic kwenye mimea yako ya matumbo na maambukizi yanayowezekana ndani ya familia yako. Matokeo ya kipimo yatakuwa ndani ya siku 5 hadi 8 na itamfahamisha daktari wako ikiwa dawa inayotumiwa inafaa au ikiwa kuna haja ya kubadili matibabu. Kwa makubaliano yako, matokeo ya uchambuzi wa mikrobiolojia yatawasilishwa kwako na kwa daktari wako. Una huru wa kurudi hospitalini kupata maelezo kuhusu matokeo hayo na matibabu yako.

Faida kuu ni kwamba utasaidia kuboresha uelewaji wa upinzani wa antimicrobial, maambukizi yake na kuendelea katika jamii.

Matokeo ya utafiti huu yanaweza kusaidia kuboresha matibabu ya antibiotic ya maambukizo na bakteria sugu katika siku zijazo.

Changamoto kuu wa ushiriki ni wakati unaohitajika kwa ukusanyaji wa sampuli, na kujibu maswali ya fomu ya utafiti.

1. **Matokeo haya yatatumika kwa njia gani?**

Matokeo ya utafiti huu yatashirikiwa na watafiti na matabibu wengine kote Ulaya na Kenya. Zitatumika kuboresha matibabu ya antibiotic na kupunguza hatari ya upinzani wa antibiotic.

1. **Je! Nitaarifiwa juu ya matokeo mapya kutoka kwa utafiti?**

Daktari atakujulisha matokeo ya sampuli zako. Mara tu utafiti ukikamilika na kuchambuliwa, utaarifiwa pia matokeo kwa maandishi.

1. **Je! Ushiriki wangu ni wa siri?**

Takwimu zako zote za kibinafsi na za matibabu zilizokusanywa wakati wa utafiti zitachukuliwa kama siri. Wachunguzi tu wa utafiti huu na wawakilishi wa Kamati ya Maadili ya Chuo Kikuu cha Kisii wameidhinishwa kuchunguza rekodi za matibabu za wagonjwa. Uchambuzi unaweza kuchapishwa katika majarida ya kisayansi lakini jina lako halitaonekana kwenye majarida haya. Watu wote wanaoweza kupata rekodi zako za matibabu watafungwa na usiri na utambulisho wako hautafunuliwa kamwe.

Daktari wako anayehudhuria atajulishwa juu ya ushiriki wako katika utafiti huu. Ikiwa ni lazima, ataweza kutupatia habari juu ya ufuatiliaji wako.

Walakini, daktari wako hataona majibu yako ya fomu ya utafiti. Rekodi za utafiti zitahifadhiwa kwa miaka 10. Habari zote zitahifadhiwa kwa siri na sampuli za kibaolojia zitashughulikiwa na maabara zinazoshiriki kwenye utafiti pekee.

Hakuna majaribio mengine yatakayofanyika kwenye sampuli za kibaolojia isipokuwa zile zilizopangwa kwa utafiti huu. Maelezo yako ya mawasiliano yatapewa tu kwa wachunguzi wa utafiti wa karibu.

1. **Je! Kutakuwa na gharama yoyote ya kibinafsi au ya bima ya afya inayohusishwa na ushiriki wangu katika utafiti huu?**

Hakutakuwa na gharama ya ziada kwako kushiriki katika utafiti huu. Upimaji wa kinyesi kwa uwepo wa bakteria sugu hautakuwa na gharama kwako.

Asante kwa kuchukua muda kusoma karatasi hii ya habari na kwa kuzingatia kushiriki katika utafiti huu. Ikiwa unataka kushiriki, daktari wako atakuuliza utie saini fomu ya idhini.

**Maelezo ya Mawasiliano**

Ikiwa una maswali zaidi, tafadhali jisikie huru kuwasiliana na wachunguzi wakuu wa utafiti:

Chuo Kikuu cha Kisii

Shule ya sayansi za Afya

Sanduku La Posta 408-40200

Kando ya barabara ya Kisii-Kilgoris

Kisii

Dk Wycliffe Mogoa

Barua pepe: drwmogoa@gmail.com au [som@kisiiuniversity.ac.ke](mailto:som@kisiiuniversity.ac.ke)

Dk Oscar Asanya Nyangiri

Barua pepe: oasanya@gmail.com

Dk Eric Omwenga Omori

Barua pepe: omorieric@kisiiuniversity.ac.ke au [omorieric@gmail.com](mailto:omorieric@gmail.com)

Maswali kuhusu haki zako kama mshiriki: Unaweza kuwasiliana na Kamati ya Maadili na Utafiti wa Taasisi (MTRH // MU-IREC) 0787723677 au barua pepe irec@mtrh.go.ke au irecoffice@gmail.com. MTRH // MU-IREC ni kikundi cha watu ambao hupitia tafiti za usalama na kulinda haki za washiriki.

**Fomu Ya Ukubali Wa Taarifa**

**Sehemu I: Ithini Kushiriki Utafiti**

| **Utafiti wa KISII ONEHEALTH AMR**  **(Konserve-AB)** | |
| --- | --- |
| **Mahali pa utafiti: Shule ya Utabibu, Chuo Kikuu cha Kisii,**  **Kisii, Kenya** | |
| **Mchunguzi / Mpelelezi Mwenza:** | |
| **Mgonjwa:**  Jina la ukoo, Jina la kwanza | |
| **Tarehe ya kuzaliwa:**\|___\|___\| \|___\|___\| \|__\|__\|___\|___\| | Jinsia :  O Mwanaume O Mwanamke |

- Natangaza kwamba nimearifiwa kwa mdomo na kwa maandishi na mfanyakazi wa afya anayesaini kuhusu utafiti uliotajwa hapo juu.
- Ninathibitisha kuwa nimesoma na kuelewa habari kwa washiriki ambayo nilipewa (toleo la 1 la 28.03.2021) na kwamba nimepata nafasi ya kuuliza maswali. Nimepokea nakala ya fomu ya habari na idhini.
- Ninaelewa kuwa ushiriki wangu ni wa hiari na kwamba niko huru kujiondoa kwenye utafiti wakati wowote, bila kutoa sababu yoyote, bila kuathiri huduma yangu ya matibabu au haki za kisheria.
- Ninathibitisha kuwa nimekuwa na wakati wa kutosha kufanya uamuzi kuhusu ushiriki wangu katika utafiti
- Nimearifiwa juu ya wajibu wa Chuo Kikuu cha Kisii kunilipa fidia kwa uharibifu wowote ambao unaweza kutokea wakati wa utafiti huu.
- Ninaelewa kuwa sehemu zingine za faili yangu ya matibabu kuhusiana na utafiti zitachunguzwa na watafiti katika Hospitali za Chuo Kikuu cha Kisii au na mamlaka ya udhibiti, wakati wanaheshimu usiri. Ninatoa idhini kwa watu hawa kupata rekodi zangu, pamoja na habari yangu ya mawasiliano, na kuwasiliana na wachunguzi.
- Ninatoa idhini yangu kwa wachunguzi kupata habari za matibabu kuhusu mimi kutoka kwa daktari wangu anayenitibu.
- Ninatoa idhini yangu kushiriki katika utafiti uliotajwa hapo juu.
- Ninakubali kinyesi changu kupimwa kwa upinzani wa madawa pekee
- Ninakubali kinyesi changu kifanyiwe majaribio ya ukinzani wa madawa na pia ziara ya nyumbani ili kukusanya sampuli za mazingira

| Mahali, Tarehe | Sahihi ya mgonjwa |
| --- | --- |
| Mahali, Tarehe | Jina na sahihi ya Mtafiti mkuu/ Mtafiti mwenza |

**Ikiwa hajui kusoma na kuandika**

*Shahidi anayesoma lazima asaini (ikiwezekana, mtu huyu anapaswa kuchaguliwa na mshiriki na haipaswi kuwa na uhusiano na timu ya utafiti). Washiriki ambao hawajui kusoma na kuandika wanapaswa kujumuisha alama zao za vidole pia*

Nimeshuhudia usomaji sahihi wa fomu ya idhini kwa mshiriki anayeweza kushiriki, na mtu huyo amepata nafasi ya kuuliza maswali. Ninathibitisha kuwa mtu huyo ametoa idhini kwa uhuru.

***Chapisha jina la shahidi _____________________ NA Uchapishaji wa kidole gumba cha mshiriki***

***Sahihi ya shaidi ______________________***

**Tarehe ________________________**

**Siku/Mwezi/Mwaka**

**Sehemu II. Fomu ya Idhini iliyojulishwa kuhusu uhifadhi na matumizi ya baadaye ya sampuli Zisizotumiwa**

Ikiwa sampuli ya kinyesi nilichotoa kwa mradi huu wa utafiti haitumiki au imebaki wakati mradi umekamilika (Tiki chaguo moja kutoka kwa kila sanduku zifuatazo)

- Nataka sampuli yangu ya kinyesi iharibiwe mara moja
- Nataka sampuli yangu ya kinyesi iharibiwe baada ya miaka ____.
- Natoa idhini sampuli yangu ihifadhiwe kwa muda mrefu

NA (ikiwa sampuli itahifadhiwa)

- Ninatoa idhini ya sampuli yangu ya kinyesi kuhifadhiwa na kutumiwa katika utafiti wa baadaye lakini tu kwenye somo sawa na mradi wa utafiti wa sasa: KONSERVE-AB
- Ninatoa ruhusa yangu kwa sampuli yangu ya kinyesi kuhifadhiwa na kutumiwa katika utafiti wa siku zijazo wa aina yoyote ambayo imeidhinishwa vyema
- Ninatoa ruhusa kwa sampuli yangu ya kinyesi kuhifadhiwa na kutumiwa katika utafiti wa siku zijazo isipokuwa kwa utafiti kuhusu ________________________

NA

- Nataka kitambulisho changu kiondolewe kwenye sampuli yangu ya kinyesi.
- Nataka kitambulisho changu kihifadhiwe na sampuli yangu ya kinyesi.

Nimesoma habari, au imesomwa kwangu. Nimepata nafasi ya kuuliza maswali juu yake na maswali yangu yamejibiwa kwa kuridhika kwangu. Ninakubali kwa hiari yangu kuwa na sampuli zangu zimehifadhiwa kwa njia na kwa kusudi lililoonyeshwa hapo juu.

Chapisha Jina la Mshiriki ______________Saini ya Mshiriki ___________________

Tarehe ___________________________Siku / mwezi / mwaka

**Ikiwa hajui kusoma na kuandika**

*Shahidi anayesoma lazima asaini (ikiwezekana, mtu huyu anapaswa kuchaguliwa na mshiriki na haipaswi kuwa na uhusiano na timu ya utafiti). Washiriki ambao hawajui kusoma na kuandika wanapaswa kujumuisha alama zao za vidole pia.*

Nimeshuhudia usomaji sahihi wa fomu ya idhini kwa mshiriki anayeweza kushiriki, na mtu huyo amepata nafasi ya kuuliza maswali. Ninathibitisha kuwa mtu huyo ametoa idhini kwa uhuru.

***Chapisha jina la shahidi _____________________ NA Uchapishaji wa kidole gumba cha mshiriki***

***Saini ya shahidi ______________________***

**Tarehe ________________________**

**Siku / mwezi / mwaka**

**Kauli ya mtafiti / mtu anayekubali idhini**

Nimesoma kwa usahihi karatasi ya habari kwa mshiriki anayeweza kushiriki, na kwa kadiri ya uwezo wangu nilihakikisha kwamba mshiriki anaelewa kuwa sampuli itahifadhiwa kwa matumizi ya baadaye kulingana na chaguo la mshiriki aliyechaguliwa hapo juu.

Ninathibitisha kuwa mshiriki alipewa nafasi ya kuuliza maswali juu ya hali na namna ya kuhifadhi sampuli, na maswali yote yaliyoulizwa na mshiriki yamejibiwa kwa usahihi na kwa kadiri ya uwezo wangu. Ninathibitisha kwamba mtu huyo hajalazimishwa kutoa idhini, na idhini hiyo imepewa kwa hiari na kwa hiari.

**Nakala ya fomu hii ya idhini imetolewa kwa mshiriki.**

**Chapisha Jina la Mtafiti / mtu anayechukua idhini __________________________**

**Tarehe ___________________________**

**Siku / mwezi / mwaka**

## ****Assent form 12 to 17 Years****

### English Version

**Information for participants**

**This Informed assent form has two parts:**

• Part I: Information Sheet [to share information about the study with you]

• Part II: Certificate of Consent [for your name if you choose to participate]

**Important things to know…**

- You get to decide if you want to take part.
- You can say ‘No’ or you can say ‘Yes’.
- No one will be annoyed if you say ‘No’.
- If you say ‘Yes’, you can always say ‘No’ later.
- You can say ‘No’ at any time.
- We will still take good care of you no matter what you decide.

**Why are we doing this study?**

We are doing this research to find out more about the germs that cause your illness. Sometimes they change and are not able to be killed by the medicine we use. We want to test to find out what has changed inside them. If we find the factors that make germs not to be killed we will visit your home to check if animals around or water also have the same changes.

**What will happen if I join this research?**

If you agree to be in the study, we will ask you to do the following:

- - stool sample: We will give you a small container and instructions to collect your stool. Amount to collect is the size of a peanut.
  - Questions: We will read to you questions on a piece of paper. You can mark your answers on the paper or say to us what is the answer.
  - Allow use of your treatment records: We will look at your records for this hospital visit.
  - Visit at your home: In case we find factors in your stool causing the germs not to be killed by medicine, we will visit your home to collect domestic animal droppings, water and soil to test them as well.

**Will I be hurt if I join this research?**

Although it is not painful, you may feel uncomfortable to give a stool sample or you may find questions hard to answer. Some of your time will be used working with researchers. We will try to make sure that no bad things happen. We will give you instructions to help collect your stool. You can say ‘no’ to what we ask you to do for the research at any time and we will stop.

**Could the research help me?**

Being in this research may not help you immediately. The results of the test will be within 5 to 8 days and will inform your doctor on whether to change the medicine in case it is not working. We will find out what are the factors that make the medicine not to work. We hope to learn something from this research that will allow use of medicines in the community for a long time.

**What else should I know about this research?**

If you do not want to be in the study, you do not have to agree.

It is also OK to say yes and change your mind later. You can stop being in the research at any time. If you want to stop, please tell the research doctors.

You will not be paid to be in the study.

You can ask questions any time. You can talk to

Dr Wycliffe Mogoa

Email: [drwmogoa@gmail.com](mailto:drwmogoa@gmail.com) or [som@kisiiuniversity.ac.ke](mailto:som@kisiiuniversity.ac.ke)

Dr Oscar Asanya Nyangiri

Email: [oasanya@gmail.com](mailto:oasanya@gmail.com)

Dr Eric Omwenga Omori

E-mail: [omorieric@kisiiuniversity.ac.ke](mailto:omorieric@kisiiuniversity.ac.ke) or [omorieric@gmail.com](mailto:omorieric@gmail.com)

Kisii University

School of Health sciences

P. O. Box 408-40200

Along Kisii-Kilgoris road

Kisii

Questions about your rights: You may contact the Institutional Ethics and Research Committee (MTRH/MU-IREC) 0787723677 or email irec@mtrh.go.ke or [irecoffice@gmail.com](mailto:irecoffice@gmail.com). The MTRH/MU-IREC is a group of people that review studies for safety and to protect the rights of those who take part.

Ask us any questions you have. Take the time you need to make your choice.

**Part II: Giving your Assent to participate**

Writing your name below shows we have talked about the research and you agree to take part. We will also write our name.

- I agree to my stool being tested for resistance to antibiotics only
- I agree to my stool being tested for resistance to antibiotics and also a home visit to collect environmental samples

| Place, date | Signature of the patient |
| --- | --- |
| Place, date | Name and signature of the Principal Investigator / Co-investigator |

**If you cannot read**

*A person who can read and write must sign as a witness. If possible, this person should be chosen by the participant and should have no connection to the research team. Participants should include their thumb-print.*

I have witnessed the correct reading of the consent form to the potential participant or their parent. The individual has had the opportunity to ask questions. I confirm that the individual has given consent freely.

***Name of witness_____________________ AND Thumb print of participant***

***Signature of witness ______________________***

**Date ________________________**

**Day/month/year**

**Part III. Informed Consent/assent for Storage and future use of samples**

If the stool sample I have provided for this research project is unused or leftover when the study is completed (Tick **one** choice from each of the following boxes)

- I wish my stool sample to be destroyed immediately.
- I want my stool sample to be destroyed after ____ years.
- I give permission for my stool sample to be stored indefinitely

AND (if the sample is to be stored)

- I give permission for my stool sample to be stored and used in future research but only on the same subject as the current research project: KONSERVE-AB
- I give my permission for my stool sample to be stored and used in future research of any type which has been properly approved
- I give permission for my stool sample to be stored and used in future research except for research about __________________________

AND

- I want my identity to be removed from my stool sample.
- I want my identity to be kept with my stool sample.

I have read the information, or it has been read to me. I have had the chance to ask questions about it and my questions have been answered to my satisfaction. I agree voluntarily to have my samples stored for the reasons shown above.

Print Name of Participant__________________

Signature of Participant ___________________

Date ___________________________

Day/month/year

**If you cannot read or write**

*A person who can read and write will witness and sign (if possible, this person should be selected by the participant and should have no connection to the research team). Participants who are illiterate should include their thumb-print as well.*

I have witnessed the correct reading of the consent form to the potential participant. The individual has had the chance to ask questions. I confirm that the individual has given consent freely.

***Print name of witness_____________________ AND Thumb print of participant***

***Signature of witness ______________________***

**Date ________________________**

**Day/month/year**

**Statement by researcher or person taking the consent**

I have correctly read out the information sheet to the potential participant. I have done my best to ensure that the participant understands that the sample will be stored for future use. This will be according to the participant’s choice ticked above.

I confirm that:

- The participant was given a chance to ask questions about the kind of storage of samples.
- All questions asked by the participant have been answered correctly and to the best of my ability. The individual has not been forced into agreeing to participate. The agreement has been given freely and voluntarily.

 A copy of this informed consent form has been provided to the participant.

Name of Researcher/person taking the consent________________________

Signature of Researcher /person taking the consent__________________________

Date ___________________________

Day/month/year

### Swahili Version

**Fomu ya idhini ya:** Watoto (miaka 12 hadi 17) katika hospitali ya Kisii Teaching na Rufaa ambao wana homa, kuhara na / au kutapika

**Fomu hii ya idhini ya habari ina sehemu mbili:**

- Sehemu ya 1: Karatasi ya Habari [Kukupa habaria]
- Sehemu ya II: Hati ya idhini [ya jina lako ikiwa utachagua kushiriki]

**Mambo muhimu ya kujua…**

• Unapaswa kuamua ikiwa unataka kushiriki.

• Unaweza kusema 'Hapana' au unaweza kusema 'Ndio'.

• Hakuna mtu atakayekasirika ukisema 'Hapana'.

• Ikiwa unasema 'Ndio', unaweza kusema "Hapana" baadaye.

• Unaweza kusema 'Hapana' wakati wowote.

• Bado tutakujali vizuri bila kujali unaamua nini.

**Kwa nini tunafanya utafiti huu?**

Tunafanya utafiti huu ili kujua zaidi juu ya vidudu vinavyosababisha ugonjwa wako. Wakati mwingine hubadilika na hawawezi kuuawa na dawa tunayotumia. Tunataka kujaribu kujua ni nini kimebadilika ndani yao. Ikiwa tutapata sababu zinazofanya vijidudu visiuawe tutatembelea nyumba yako kuangalia ikiwa wanyama karibu au maji pia wana mabadiliko sawa.

**Je! Nini itatokea nikijiunga na utafiti huu?**

Ikiwa unakubali kuwa katika utafiti, tutakuuliza ufanye yafuatayo:

- - sampuli ya kinyesi: Tutakupa kontena dogo na maagizo ya kukusanya kinyesi chako. Kiasi cha kukusanya ni saizi ya karanga.
  - Maswali: Tutakusomea maswali kwenye karatasi. Unaweza kuweka alama kwenye majibu yako au utuambie jibu ni nini.
  - Ruhusu matumizi ya rekodi zako za matibabu: Tutaangalia rekodi zako kwa ziara hii ya hospitali.
  - Matembezi nyumbani kwako: Endapo tutapata sababu kwenye kinyesi chako zinazosababisha vijidudu kutouliwa na dawa, tutatembelea nyumba yako kukusanya kinyesi cha wanyama wa kufugwa, maji na udongo ili kuwafanyia vipimo kutafuta vidudu sugu.

**Je! Nitaumia ikiwa nitajiunga na utafiti huu?**

Ingawa sio chungu, unaweza kuhisi wasiwasi kutoa sampuli ya kinyesi au unaweza kupata maswali magumu kujibu. Wakati wako utatumika kufanya kazi na watafiti. Tutajaribu kuhakikisha kuwa hakuna mambo mabaya yanayotokea. Tutakupa maagizo ya kusaidia kukusanya kinyesi chako. Unaweza kusema 'hapana' kwa yale tunakuuliza ufanye kwa utafiti wakati wowote na tutaacha.

**Je! Utafiti unaweza kunisaidia?**

Kuwa katika utafiti huu hakuwezi kukusaidia mara moja. Matokeo ya kipimo yatakuwa ndani ya siku 5 hadi 8 na yatamwarifu daktari wako juu ya kubadilisha dawa ikiwa haifanyi kazi. Tutagundua ni mambo gani ambayo hufanya dawa hiyo isifanye kazi. Tunatarajia kujifunza kitu kutoka kwa utafiti huu ambao utaruhusu matumizi ya dawa katika jamii kwa muda mrefu.

**Ni nini kingine nipaswa kujua kuhusu utafiti huu?**

Ikiwa hautaki kuwa kwenye utafiti, sio lazima ukubali. Ni sawa pia kusema ndio na ubadilishe mawazo yako baadaye. Unaweza kuacha kuwa katika utafiti wakati wowote. Ikiwa unataka kuacha, tafadhali waambie madaktari wa utafiti. Hautalipwa kuwa katika utafiti huu.

Unaweza kuuliza maswali wakati wowote. Unaweza kuzungumza na

Dr Wycliffe Mogoa

Email: [drwmogoa@gmail.com](mailto:drwmogoa@gmail.com) or [som@kisiiuniversity.ac.ke](mailto:som@kisiiuniversity.ac.ke)

Dr Oscar Asanya Nyangiri

Email: [oasanya@gmail.com](mailto:oasanya@gmail.com)

Dr Eric Omwenga Omori

E-mail: [omorieric@kisiiuniversity.ac.ke](mailto:omorieric@kisiiuniversity.ac.ke) au [omorieric@gmail.com](mailto:omorieric@gmail.com)

Anwani

Chuo kikuu cha Kisii

Shule ya sayansi za Afya

Sanduku La Posta 408-40200

Barabara ya Kisii-Kilgoris

Kisii

Maswali kuhusu haki zako: Unaweza kuwasiliana na Kamati ya Maadili na Utafiti wa Taasisi (MTRH / MU-IREC) 0787723677 au barua pepe irec@mtrh.go.ke au irecoffice@gmail.com. MTRH / MU-IREC ni kikundi cha watu ambao hupitia utafitii ili kuhakikisha usalama na kulinda haki za wale wanaoshiriki.

Tuulize maswali yoyote unayo. Chukua muda unahitaji kufanya uchaguzi wako.

**Sehemu ya II: Kutoa Kibali chako kushiriki**

Kuandika jina lako hapa chini kunaonyesha tumezungumza juu ya utafiti huo na unakubali kushiriki. Tutaandika pia jina letu.

- Ninakubali kinyesi changu kupimwa kwa upinzani wa madawa pekee
- Ninakubali kinyesi changu kifanyiwe majaribio ya ukinzani wa madawa na pia ziara ya nyumbani ili kukusanya sampuli za mazingira

| Mahali, tarehe | Saini ya mgonjwa |
| --- | --- |
| Mahali, tarehe | Jina na saini ya Mchunguzi / Mchunguzi Mkuu |

**Ikiwa huwezi kusoma**

*Mtu anayeweza kusoma na kuandika lazima atie saini kama shahidi. Ikiwezekana, mtu huyu anapaswa kuchaguliwa na mshiriki na haipaswi kuwa na uhusiano na timu ya utafiti. Washiriki wanapaswa kujumuisha uchapishaji wao wa kidole gumba.*

Nimeshuhudia usomaji sahihi wa fomu ya idhini kwa mshiriki anayeweza kushiriki au mzazi wao. Mtu huyo amekuwa na nafasi ya kuuliza maswali. Ninathibitisha kuwa mtu huyo ametoa idhini kwa uhuru.

***Jina la shahidi_____________________ NA Uchapishaji wa kidole gumba cha mshiriki***

***Saini ya shahidi ______________________***

**Tarehe ________________________**

**Siku/Mwezi/Mwaka**

**Sehemu ya III. Idhini / taarifa ya kuhifadhiwa na utumiaji wa sampuli baadaye**

Ikiwa sampuli ya kinyesi nilichotoa kwa mradi huu wa utafiti haitumiki au imebaki wakati utafiti umekamilika (Tiki chaguo moja kutoka kwa kila sanduku lifuatalo)

- Nataka sampuli yangu ya kinyesi iharibiwe mara moja.
- Ninataka sampuli yangu ya kinyesi iharibiwe baada ya miaka ____.
- Ninatoa ruhusa kwa sampuli yangu ya kinyesi kuhifadhiwa bila kikomo

NA (ikiwa sampuli itahifadhiwa)

- Ninatoa ruhusa kwa sampuli yangu ya kinyesi kuhifadhiwa na kutumiwa katika utafiti wa baadaye lakini tu kwenye somo sawa na mradi wa sasa wa utafiti: KONSERVE-AB
- Ninatoa ruhusa yangu kwa sampuli yangu ya kinyesi kuhifadhiwa na kutumiwa katika utafiti wa baadaye wa aina yoyote ambayo imeidhinishwa vyema
- Ninatoa ruhusa kwa sampuli yangu ya kinyesi kuhifadhiwa na kutumiwa katika utafiti wa baadaye isipokuwa kwa utafiti kuhusu ________________________

NA

- Nataka kitambulisho changu kiondolewe kwenye sampuli yangu ya kinyesi.
- Nataka kitambulisho changu kihifadhiwe na sampuli yangu ya kinyesi.

**Nimesoma habari, au imesomwa kwangu. Nimepata nafasi ya kuuliza maswali juu yake na maswali yangu yamejibiwa kwa kuridhika kwangu. Ninakubali kwa hiari kuhifadhi sampuli zangu kwa sababu zilizoonyeshwa hapo juu.**

Jina la mshiriki__________________

Saini ya mshiriki ___________________

Tarehe ___________________________

Siku/Mwezi/Mwaka

**Ikiwa hujui kusoma na kuandika**

*Mtu anayeweza kusoma na kuandika lazima atie saini kama shahidi. Ikiwezekana, mtu huyu anapaswa kuchaguliwa na mshiriki na haipaswi kuwa na uhusiano na timu ya utafiti. Washiriki wanapaswa kujumuisha uchapishaji wao wa kidole gumba.*

Nimeshuhudia usomaji sahihi wa fomu ya idhini kwa mshiriki anayeweza kushiriki au mzazi wao. Mtu huyo amekuwa na nafasi ya kuuliza maswali. Ninathibitisha kuwa mtu huyo ametoa idhini kwa uhuru.

***Jina la shahidi_____________________ NA Uchapishaji wa kidole gumba cha mshiriki***

***Saini ya shahidi ______________________***

**Tarehe ________________________**

**Siku/Mwezi/Mwaka**

**Kauli ya mtafiti au mtu anayechukua idhini**

Nimesoma kwa usahihi karatasi ya habari kwa mshiriki anayeweza. Nimefanya bidii kuhakikisha kuwa mshiriki anaelewa kuwa sampuli itahifadhiwa kwa matumizi ya baadaye. Hii itakuwa kulingana na chaguo la mshiriki aliyechaguliwa hapo juu.

Ninathibitisha kuwa:

- Mshiriki alipewa nafasi ya kuuliza maswali juu ya aina ya uhifadhi wa sampuli.
- Maswali yote yaliyoulizwa na mshiriki yamejibiwa kwa usahihi na kwa uwezo wangu wote. Mtu huyo hajalazimishwa kukubali kushiriki.
- Makubaliano hayo yametolewa kwa hiari na kwa hiari.

Nakala ya fomu hii ya idhini ya habari imetolewa kwa mshiriki.

**Jina la mtafiti/mtu anayechuckua idhini________________________**

**Saini ya Mtafiti / mtu anayechukua idhini __________________________**

**Tarehe ___________________________**

**Siku/Mwezi/Mwaka**

## **Parental Permission Form**

### English Version

**Information to Participants**

**What is this form is about?**

You have the option of allowing your child to join a research study. This is a parental permission form. The goal of this form is to give you information about what would happen in the study if you choose to have your child take part and to help you decide if you want your child to be in the study. It provides a summary of the information the research team will discuss with you. If you decide that your child can take part in this study, you would sign this form to confirm your decision. If you sign this form, you will receive a signed copy for your records. You can take notes, write questions or highlight any part of this form.

**What you should know about this study**

- This form explains what would happen if your child joins this research study.
- Please read it carefully. Take as much time as you need.
- Please ask the research team questions about anything that is not clear.
- You can ask questions about the study any time.
- If you choose not to have your child join the study, it will not affect their care at Kisii Teaching and Referral hospital.
- If you say ‘Yes’ now, you can still change your mind later.
- You can choose to have your child leave the study at any time.
- Your child would not lose benefits or be penalized if you decide not to have your child take part in the study or leave the study later.

**Why do I have the option of having my child joining the study?**

You have the option of having your child join the study for one of two reasons.

He/she is in the hospital with fever and diarrhoea and is suspected to have bacterial infection in the gut.

**Who cannot participate?**

A child or adult may not participate if they live more than 25km from Kisii. In addition, those who refuse to give consent or have been subjected to antibiotic usage will be excluded.

**Can my child be excluded from the study after I give my consent?**

The study investigators can decide at any time to stop your child’s participation if it is in your child’s best result to do so, without having to obtain your prior consent. You will be informed of the reason for stopping.

**Does my child have to take part in this study and what are my child’s rights?**

Your child’s participation in this study is completely voluntary. Not taking part in the study will not affect his/her future medical care. The same principle applies if you take back your initial consent for your child after starting the study. You can therefore withdraw your child’s participation at any time without having to justify your decision. In the event of revocation, the data collected up to that point will continue to be used.

**What should I do if I agree for my child to participate in this study?**

Participants will have the tests described below.

If you agree for your child to take part in this study, the study investigator will ask you to sign a consent form to confirm that you understand the study and agree for their participation. Your child’s participation in the study will be recorded in your computerized care record. You will not receive any experimental treatment.

We will ask you questions about your child’s illness, medical history, contact with animals and dietary preferences. Your child will be asked to provide a stool sample. If the child’s sample tests positive for the resistant Enterobacteriaceae bacteria, environmental samples such as water sample, soil, animal stool sample if any will be collected from your household.

**Will these tests cause any discomfort?**

Stool sampling does not cause any physical discomfort.

**What happens to the samples once they are collected?**

A study investigator will collect the samples from your child. They will be centralized, analyzed at the Kisii University school of health sciences laboratory and used for research purposes. They will be treated in a strictly confidential and anonymous manner; personal identifiers will be replaced by a code. The only people who have access to this code are the principal investigator of the study, Dr Wycliffe Mogoa, and his collaborators who are in charge of the study. They will then be kept for 10 years. You have the right to see the data and to have the samples destroyed. Any resistant bacteria found in any of the samples will be isolated and compared with each other in the German collaborative University, the Hohenheim University.

**What are the benefits and disadvantages of the study?**

Participation in the study does not create any direct benefit for your child apart from the knowledge of the antibiotic resistance in your intestinal flora and the possible transmission within your family. With your agreement, the results of the microbiological analysis will be communicated to you, your child and your doctor.

The main benefit is that it will help improve the understanding of antimicrobial resistance, its transmission and persistence. The results of this research may help to improve antibiotic treatment of infections with resistant bacteria in the future.

The main disadvantage of participation is the time required for sample collection, and the questionnaire.

**What will these results be used for?**

The results of this study will be shared with other researchers and physicians in Kenya and Europe. They will be used to improve antibiotic treatment and minimize the risk of antibiotic resistance.

**Will I be informed of new findings from the study?**

The doctor will inform you of the results of your child’s samples. Once the study is completed and analysed, you will also be informed of the results in writing.

**Is my child’s participation confidential?**

All of your child’s personal and medical data collected during the study will be treated as confidential. Only the investigators of this study, the representatives of the Ethics Committee of the Kisii University are authorized to examine the patients' medical records. Analyses may be published in scientific journals but your name will not appear in these journals. All persons having access to your medical records will be bound by confidentiality and your identity will never be revealed.

Your attending physician will be informed of your participation in this study. If necessary, he or she will be able to provide us with information about your follow-up. However, your doctor will not see your answers to the questionnaire. Study records will be kept for 10 years. All information will be kept strictly confidential and microbiological samples will be processed exclusively by the laboratories participating in the study. No other tests will be performed on the microbiological samples other than those planned for this study. Your contact information will only be given to the local study investigators.

**Will there be any personal costs associated with my child’s participation in this study?**

There will be no additional cost to you for participating in this study. The stool testing for presence of resistant bacteria will be of no cost for you.

Thank you for taking the time to read this information sheet and for considering participating in this study. If you want your child to participate, your doctor will ask you to sign a consent form on behalf of the child.

**Contact Information for questions about the study**

If you have any more questions, please feel free to contact the principal investigators of the study:

Kisii University

School of Health sciences

P. O. Box 408-40200

Along Kisii-Kilgoris road

Kisii

Dr Wycliffe Mogoa

Email: [drwmogoa@gmail.com](mailto:drwmogoa@gmail.com) or [som@kisiiuniversity.ac.ke](mailto:som@kisiiuniversity.ac.ke)

Dr Oscar Asanya Nyangiri

Email: [oasanya@gmail.com](mailto:oasanya@gmail.com)

Dr Eric Omwenga Omori

E-mail: [omorieric@kisiiuniversity.ac.ke](mailto:omorieric@kisiiuniversity.ac.ke) or [omorieric@gmail.com](mailto:omorieric@gmail.com)

Questions about your child’s rights as a participant: You may contact the Institutional Ethics and Research Committee (MTRH//MU-IREC) 0787723677 or email irec@mtrh.go.ke or [irecoffice@gmail.com](mailto:irecoffice@gmail.com). The MTRH//MU-IREC is a group of people that review studies for safety and to protect the rights of participants.

**Informed Consent Form**

**Part I : Consnet to Participate in the Study**

| **KISII Onehealth amr project**  **(Konserve-AB)** | |
| --- | --- |
| **Location of the study: Kisii University School of Medicine,**  **Kisii, Kenya** | |
| **Investigator/ Co-investigator:** | |
| **Patient :**  Surname, Name | |
| **Date of birth:** \|___\|___\| \|___\|___\| \|__\|__\|___\|___\| | Sex : O male O female |

- I declare that I have been informed orally and in writing by the signing health worker about the above mentioned study.
- I confirm that I have read and understood the information to participants that was given to me (version 1. of 28.03.2021) and that I have had the opportunity to ask questions. I have received a copy of the information and consent form.
- I understand that my child’s participation is voluntary and that I am free to withdraw my child from the study at any time, without giving any reason, without affecting his/her medical care or legal rights.
- I confirm that I have had sufficient time to make a decision regarding my child’s participation in the study
- I have been informed of the obligation of the Kisii University to compensate me for any damage that may occur in the course of this study.
- I understand that certain parts of my child’s medical file in relation to the study will be examined by researchers at the University Hospitals of Kisii or by regulatory authorities, while respecting confidentiality. I give permission to these individuals to have access to my child’s records, including my child’s contact information, and to be contacted by the investigators.
- I give my consent for the investigators to obtain medical information about my child from his/her treating physician.
- I give consent for my child to participate in the above mentioned study.

| Place, date | Signature of the parent |
| --- | --- |
| Place, date | Name and signature of the Principal Investigator / Co-investigator |

**If illiterate**

*A literate witness must sign (if possible, this person should be selected by the participant and should have no connection to the research team). Participants who are illiterate should include their thumb-print as well.*

I have witnessed the accurate reading of the consent form to the potential participant’s parent/guardian, and the individual has had the opportunity to ask questions. I confirm that the individual has given consent freely.

***Print name of witness_____________________ AND Thumb print of participant***

***Signature of witness ______________________***

**Date ________________________**

**Day/month/year**

**Part II. Informed Consent Form for Consent for Storage and Future Use of Unused Samples**

If the stool sample the child has provided for this research project is unused or leftover when the project is completed (Tick **one** choice from each of the following boxes)

- I wish my child’s stool sample to be destroyed immediately.
- I want my child’s stool sample to be destroyed after ____ years.
- I give permission for my child’s stool sample to be stored indefinitely

AND (if the sample is to be stored)

- I give my permission for my child’s stool sample to be stored and used in future research but only in the same subject as the current research project: KONSERVE-AB
- I give permission for my child’s stool sample to be store and used in future research of any type which has been properly approved
- I give permission for my child’s stool sample to be stored and used in future research except for research about ______________________

AND

- I want my child’s identity to be removed from my stool sample.
- I want my child’s identity to be kept with my stool sample.

I have read the information, or it has been read to me. I have had the opportunity to ask questions about it and my questions have been answered to my satisfaction. I consent voluntarily to have my samples stored in the manner and for the purpose indicated above.

Print Name of Participant__________________

Signature of Participant ___________________

Date ___________________________

Day/month/year

**If illiterate**

*A literate witness must sign (if possible, this person should be selected by the participant and should have no connection to the research team). Participants who are illiterate should include their thumb-print as well.*

I have witnessed the accurate reading of the consent form to the potential participant, and the individual has had the opportunity to ask questions. I confirm that the individual has given consent freely.

*Print name of witness_____________________ AND Thumb print of participant*

*Signature of witness ______________________*

Date ________________________

Day/month/year

**Statement by the researcher/person taking consent**

I have accurately read out the information sheet to the potential participant or their parent/guardian, and to the best of my ability made sure that the parent/guardian understands that the sample will be stored for future use in accordance to the participant’s choice ticked above.

I confirm that the participant was given an opportunity to ask questions about the nature and manner of storage of the samples, and all the questions asked by the participant have been answered correctly and to the best of my ability. I confirm that the individual has not been coerced into giving consent, and the consent has been given freely and voluntarily**.** A copy of this ICF has been provided to the participant.

Print Name of Researcher/person taking the consent________________________

Signature of Researcher /person taking the consent__________________________

Date ___________________________

Day/month/year

### Swahili Version

**Habari Kwa Washiriki**

**Kichwa cha Utafiti: Kuchunguza upinzani wa antibiotic na maambukizi kwa Fomu hii inahusu nini?**

Una chaguo la kumruhusu mtoto wako ajiunge na utafiti wa utafiti. Hii ni fomu ya ruhusa ya wazazi. Lengo la fomu hii ni kukupa habari kuhusu nini kitatokea katika utafiti ikiwa utachagua mtoto wako kushiriki na kukusaidia kuamua ikiwa unataka mtoto wako awepo kwenye utafiti. Inatoa muhtasari wa habari ambayo timu ya utafiti itajadili na wewe. Ukiamua kuwa mtoto wako anaweza kushiriki katika utafiti huu, utasaini fomu hii ili kudhibitisha uamuzi wako. Ukisaini fomu hii, utapokea nakala iliyosainiwa kwa kumbukumbu zako. Unaweza kuchukua maelezo, kuandika maswali au kuonyesha sehemu yoyote ya fomu hii.

**Nini unapaswa kujua kuhusu utafiti huu**

- Fomu hii inaelezea nini kitatokea ikiwa mtoto wako atajiunga na utafiti huu.
- Tafadhali isome kwa uangalifu. Chukua muda mwingi kama unahitaji.
- Tafadhali uliza timu ya watafiti maswali juu ya chochote ambacho hakieleweki.
- Unaweza kuuliza maswali juu ya utafiti wakati wowote.
- Ukichagua kutomruhusu mtoto wako ajiunge na utafiti, haitaathiri utunzaji wao katika Kisii Teaching and Referral hospital.
- Ikiwa unasema 'Ndio' sasa, bado unaweza kubadilisha mawazo yako baadaye.
- Unaweza kuchagua mtoto wako aondoke kwenye masomo wakati wowote.
- Mtoto wako hatapoteza faida au kuadhibiwa ikiwa utaamua kutomruhusu mtoto wako kushiriki katika utafiti au kuacha masomo baadaye.

**Muhtasari mfupi wa utafiti**

KONSERVE-AB ni utafiti wa kushirikiana ambao unakusudia kujua ikiwa mtoto wako ana aina ya bakteria ambayo haitii dawa, na kuiunganisha na mazingira ya kaya kutafuta uambukizo unaowezekana kati ya wagonjwa wanaogunduliwa na bakteria hawa. Katika kaya hii itajumuisha kinyesi cha wanyama, udongo na maji.

Imepangwa kujumuisha wagonjwa 200 wenye homa na kuhara. Hii ni utafiti wa uchunguzi ambapo hakuna dawa ya majaribio itapewa.

Washiriki wataulizwa kukamilisha dodoso. Sampuli za mikrobiolojia (sampuli za kinyesi) zitakusanywa hospitalini. Kushiriki katika utafiti huu ni kwa hiari kabisa.

**Kwa nini nina chaguo la mtoto wangu kujiunga na utafiti?**

Una chaguo la kumruhusu mtoto wako ajiunge na utafiti kwa sababu moja kati ya mbili.

Yuko hospitalini na homa na kuhara na anashukiwa kuwa na maambukizo ya bakteria kwenye utumbo.

**Nani hawezi kushiriki?** Mtoto au mtu mzima hatashiriki ikiwa anaishi zaidi ya kilomita 25 kutoka Kisii. Kwa kuongezea, wale ambao wanakataa kutoa idhini pia hawatashiriki.

**Je! Mtoto wangu anaweza kutengwa kwenye utafiti baada ya kutoa idhini yangu?**

Wachunguzi wa utafiti wanaweza kuamua wakati wowote kuzuia ushiriki wa mtoto wako ikiwa ni katika matokeo bora ya mtoto wako kufanya hivyo, bila kupata idhini yako ya awali. Utajulishwa sababu ya kuacha.

**Je! Mtoto wangu lazima ashiriki katika utafiti huu na ni haki gani za mtoto wangu?**

Ushiriki wa mtoto wako katika utafiti huu ni wa hiari kabisa. Kutokushiriki katika utafiti hakuathiri matibabu yake ya baadaye. Kanuni hiyo hiyo inatumika ikiwa utarudisha idhini yako ya kwanza kwa mtoto wako baada ya kuanza masomo. Kwa hivyo unaweza kuondoa ushiriki wa mtoto wako wakati wowote bila kuhalalisha uamuzi wako. Katika tukio la kufutwa, data iliyokusanywa hadi wakati huo itaendelea kutumiwa.

**Nifanye nini ikiwa ninakubali mtoto wangu kushiriki katika utafiti huu?**

Washiriki watakuwa na vipimo vilivyoelezewa hapa chini.

Ikiwa unakubali mtoto wako kushiriki katika utafiti huu, mchunguzi wa utafiti atakuuliza utia saini fomu ya idhini ili kuthibitisha kuwa unaelewa utafiti huo na unakubali kushiriki kwao. Ushiriki wa mtoto wako katika utafiti utarekodiwa katika rekodi yako ya utunzaji wa kompyuta. Hautapokea matibabu yoyote ya majaribio.

Tutakuuliza maswali juu ya ugonjwa wa mtoto wako, historia ya matibabu, mawasiliano na wanyama na upendeleo wa lishe. Mtoto wako ataulizwa kutoa sampuli ya kinyesi. Ikiwa sampuli za mtoto zinaonyesha dalili kwa bakteria sugu ya Enterobacteriaceae, sampuli za mazingira kama vile sampuli ya maji, mchanga, sampuli ya kinyesi cha wanyama ikiwa itakusanywa kutoka kwa boma yako.

**Je! Vipimo hivi vitasababisha usumbufu wowote?**

Sampuli ya kinyesi haisababishi usumbufu wowote wa mwili.

**Ni nini hufanyika kwa sampuli mara zinapokusanywa?**

Mchunguzi wa utafiti atakusanya sampuli kutoka kwa mtoto wako. Yatawekwa katikati, kuchambuliwa katika shule ya Chuo Kikuu cha Kisii ya maabara ya sayansi ya afya na kutumika kwa madhumuni ya utafiti. Watashughulikiwa kwa njia ya siri na isiyojulikana; vitambulisho vya kibinafsi vitabadilishwa na nambari. Watu pekee ambao wanapata msimbo huu ni mchunguzi mkuu wa utafiti huo, Dk Wycliffe Mogoa, na washirika wake ambao wanasimamia utafiti huo. Kisha watahifadhiwa kwa miaka 10. Una haki ya kuona data na kuharibiwa sampuli. Bakteria yoyote sugu inayopatikana katika sampuli yoyote itatengwa na ikilinganishwa na kila mmoja katika Chuo Kikuu cha Ushirika cha Ujerumani, Chuo Kikuu cha Hohenheim.

**Je! Ni faida na hasara gani za utafiti?**

Kushiriki katika utafiti hakuleti faida yoyote ya moja kwa moja kwa mtoto wako mbali na ujuzi wa kinga ya antibiotic kwenye mimea yako ya matumbo na maambukizi yanayowezekana ndani ya familia yako. Kwa makubaliano yako, matokeo ya uchambuzi wa viumbe vidogo yatafikishwa kwako, kwa mtoto wako na kwa daktari wako.

Faida kuu ni kwamba itasaidia kuboresha uelewa wa upinzani wa antimicrobial, maambukizi yake na kuendelea. Matokeo ya utafiti huu yanaweza kusaidia kuboresha matibabu ya antibiotic ya maambukizo na bakteria sugu katika siku zijazo.

Ubaya kuu wa kushiriki ni wakati unaohitajika kwa ukusanyaji wa sampuli, na kujibu maswali.

**Matokeo haya yatatumika kwa njia gani?**

Matokeo ya utafiti huu yatashirikiwa na watafiti wengine na waganga nchini Kenya na Ulaya. Zitatumika kuboresha matibabu ya antibiotic na kupunguza hatari ya upinzani wa antibiotic.

**Je! Nitaarifiwa juu ya matokeo mapya kutoka kwa utafiti?**

Daktari atakujulisha matokeo ya sampuli za mtoto wako. Mara tu utafiti ukikamilika na kuchambuliwa, utaarifiwa pia matokeo kwa maandishi.

**Je! Ushiriki wa mtoto wangu ni wa siri?**

Takwimu zote za kibinafsi na za matibabu za mtoto wako zilizokusanywa wakati wa utafiti zitachukuliwa kuwa za siri. Wachunguzi tu wa utafiti huu, wawakilishi wa Kamati ya Maadili ya Chuo Kikuu cha Kisii wameidhinishwa kuchunguza rekodi za matibabu za wagonjwa. Uchambuzi unaweza kuchapishwa katika majarida ya kisayansi lakini jina lako halitaonekana kwenye majarida haya. Watu wote wanaoweza kupata rekodi zako za matibabu watafungwa na usiri na utambulisho wako hautafunuliwa kamwe.

Daktari wako anayehudhuria atajulishwa juu ya ushiriki wako katika utafiti huu. Ikiwa ni lazima, ataweza kutupatia habari juu ya ufuatiliaji wako. Walakini, daktari wako hataona majibu yako kwa maswali ya kikaratasi. Rekodi za masomo zitahifadhiwa kwa miaka 10. Habari zote zitahifadhiwa kwa siri na sampuli za kibaolojia zitashughulikiwa peke na maabara zinazoshiriki kwenye utafiti. Hakuna majaribio mengine yatakayofanyika kwenye sampuli za kibaolojia isipokuwa zile zilizopangwa kwa utafiti huu. Maelezo yako ya mawasiliano yatapewa tu kwa wachunguzi wa utafiti wa karibu.

**Je! Kutakuwa na gharama zozote za kibinafsi zinazohusishwa na ushiriki wa mtoto wangu katika utafiti huu?**

Hakutakuwa na gharama ya ziada kwako kushiriki katika utafiti huu. Upimaji wa kinyesi kwa uwepo wa bakteria sugu hautakuwa na gharama kwako.

Asante kwa kuchukua muda kusoma karatasi hii ya habari na kwa kuzingatia kushiriki katika utafiti huu. Ikiwa unataka mtoto wako kushiriki, daktari wako atakuuliza utia saini fomu ya idhini kwa niaba ya mtoto.

**Maelezo ya Mawasiliano kwa maswali kuhusu utafiti**

Ikiwa una maswali zaidi, tafadhali jisikie huru kuwasiliana na wachunguzi wakuu wa utafiti:

**Anwani:** Chuo kikuu cha Kisii

Shule ya sayansi za Afya

Sanduku La Posta 408-40200

Barabara ya Kisii-Kilgoris

Kisii

Dr Wycliffe Mogoa

Email: [drwmogoa@gmail.com](mailto:drwmogoa@gmail.com) au [som@kisiiuniversity.ac.ke](mailto:som@kisiiuniversity.ac.ke)

Dr Oscar Asanya Nyangiri

Email: [oasanya@gmail.com](mailto:oasanya@gmail.com)

Dr Eric Omwenga Omori

E-mail: [omorieric@kisiiuniversity.ac.ke](mailto:omorieric@kisiiuniversity.ac.ke) au [omorieric@gmail.com](mailto:omorieric@gmail.com)

Maswali kuhusu haki za mtoto wako kama mshiriki: Unaweza kuwasiliana na Kamati ya Maadili na Utafiti wa Taasisi (MTRH // MU-IREC) 0787723677 au barua pepe irec@mtrh.go.ke au irecoffice@gmail.com. MTRH // MU-IREC ni kikundi cha watu ambao hupitia tafiti za usalama na kulinda haki za washiriki.

**Fomu Ya Ukubali Wa Taarifa**

**Sehemu 1 : Idhini Ya Kushiriki**

| **Mradi wa KISII ONEHEALTH AMR**  **(Konserve-AB)** | |
| --- | --- |
| **Mahali pa utafiti: Chuo Kikuu cha Kisii cha Tiba,**  **Kisii, Kenya** | |
| **Mchunguzi / Mpelelezi Mwenza:** | |
| **Mgonjwa:**  Jina, jina la kwanza | |
| **Tarehe ya kuzaliwa:** \|___\|___\| \|___\|___\| \|__\|__\|___\|___\| | Jinsia : O Kiume O kike |

- Natangaza kwamba nimearifiwa kwa mdomo na kwa maandishi na mfanyakazi wa afya anayesaini kuhusu utafiti uliotajwa hapo juu.
- Ninathibitisha kuwa nimesoma na kuelewa habari kwa washiriki ambayo nilipewa (toleo la 1 la 28.03.2021) na kwamba nimepata nafasi ya kuuliza maswali. Nimepokea nakala ya fomu ya habari na idhini.
- Ninaelewa kuwa ushiriki wa mtoto wangu ni wa hiari na kwamba niko huru kumtoa mtoto wangu kutoka kwa masomo wakati wowote, bila kutoa sababu yoyote, bila kuathiri huduma yake ya matibabu au haki za kisheria.
- Ninathibitisha kuwa nimekuwa na wakati wa kutosha kufanya uamuzi kuhusu ushiriki wa mtoto wangu katika utafiti
- Nimejulishwa juu ya wajibu wa Chuo Kikuu cha Kisii kunilipa fidia kwa uharibifu wowote ambao unaweza kutokea wakati wa utafiti huu.
- Ninaelewa kuwa sehemu fulani za faili ya matibabu ya mtoto wangu kuhusiana na utafiti huo itachunguzwa na watafiti katika Hospitali za Chuo Kikuu cha Kisii au na mamlaka ya udhibiti, wakati wanaheshimu usiri. Ninatoa ruhusa kwa watu hawa kupata rekodi za mtoto wangu, pamoja na maelezo ya mawasiliano ya mtoto wangu, na kuwasiliana na wachunguzi.
- Ninatoa idhini yangu kwa wachunguzi kupata habari za matibabu juu ya mtoto wangu kutoka kwa daktari wake anayemtibu.
- Ninampa idhini mtoto wangu kushiriki katika utafiti uliotajwa hapo juu.

| Mahali, tarehe | Saini ya mzazi |
| --- | --- |
| Mahali, tarehe | Jina na saini ya Mchunguzi / Mchunguzi Mkuu |

**Ikiwa hajui kusoma na kuandika**

*Shahidi anayesoma lazima asaini (ikiwezekana, mtu huyu anapaswa kuchaguliwa na mshiriki na haipaswi kuwa na uhusiano na timu ya utafiti). Washiriki ambao hawajui kusoma na kuandika wanapaswa kujumuisha alama zao za vidole pia.*

Nimeshuhudia usomaji sahihi wa fomu ya idhini kwa mzazi / mlezi wa mshiriki, na mtu huyo amepata nafasi ya kuuliza maswali. Ninathibitisha kuwa mtu huyo ametoa idhini kwa uhuru.

***Jina la shahidi _____________________ AND Uchapishaji wa kidole gumba cha mshiriki***

***Saini ya shahidi ______________________***

**Tarehe ________________________**

**Siku / mwezi / mwaka**

**Sehemu ya II. Fomu ya idhini iliyojulishwa ya idhini ya Uhifadhi na Matumizi ya Baadaye ya Sampuli zisizotumiwa**

Ikiwa sampuli ya kinyesi ambayo mtoto ametoa kwa mradi huu wa utafiti haitumiwi au imesalia wakati mradi umekamilika (Tiki chaguo moja kutoka kwa kila sanduku zifuatazo)

- Nataka sampuli ya kinyesi cha mtoto wangu iharibiwe mara moja.
- Nataka sampuli ya kinyesi cha mtoto wangu iharibiwe baada ya miaka ____.
- Natoa ruhusa kwa sampuli ya kinyesi cha mtoto wangu kuhifadhiwa kwa muda usiojulikana

NA (ikiwa sampuli itahifadhiwa)

- Ninatoa ruhusa yangu kwa sampuli ya kinyesi cha mtoto wangu kuhifadhiwa na kutumiwa katika utafiti wa baadaye lakini tu katika somo sawa na mradi wa sasa wa utafiti: KONSERVE-AB
- Natoa ruhusa kwa sampuli ya mtoto wangu kuhifadhi na kutumiwa katika utafiti wa siku zijazo wa aina yoyote ambayo imeidhinishwa vyema
- Natoa ruhusa ya sampuli ya mtoto wangu kuhifadhiwa na kutumiwa katika utafiti wa siku zijazo isipokuwa utafiti kuhusu ______________________

NA

- Nataka kitambulisho cha mtoto wangu kiondolewe kwenye sampuli ya kinyesi.
- Nataka kitambulisho cha mtoto wangu kihifadhiwe na sampuli yangu ya kinyesi.

Nimesoma habari, au imesomwa kwangu. Nimepata nafasi ya kuuliza maswali juu yake na maswali yangu yamejibiwa kwa kuridhika kwangu. Ninakubali kwa hiari yangu kuwa na sampuli zangu zimehifadhiwa kwa njia na kwa kusudi lililoonyeshwa hapo juu.

Chapisha Jina la Mshiriki __________________

Saini ya Mshiriki ___________________

Tarehe ___________________________

Siku / mwezi / mwaka

**Ikiwa hajui kusoma na kuandika**

*Shahidi anayesoma lazima asaini (ikiwezekana, mtu huyu anapaswa kuchaguliwa na mshiriki na haipaswi kuwa na uhusiano na timu ya utafiti). Washiriki ambao hawajui kusoma na kuandika wanapaswa kujumuisha alama zao za vidole pia.*

Nimeshuhudia usomaji sahihi wa fomu ya idhini kwa mshiriki anayeweza kushiriki, na mtu huyo amepata nafasi ya kuuliza maswali. Ninathibitisha kuwa mtu huyo ametoa idhini kwa uhuru.

***Jina la shahidi _____________________ NA Uchapishaji wa kidole gumba cha mshiriki***

***Saini ya shahidi ______________________***

**Tarehe ________________________**

**Siku / mwezi / mwaka**

**Kauli ya mtafiti / mtu anayekubali idhini**

- Nimesoma kwa usahihi karatasi ya habari kwa mshiriki anayeweza kushiriki au mzazi / mlezi wao, na kwa uwezo wangu wote nilihakikisha kuwa mzazi / mlezi anaelewa kuwa sampuli itahifadhiwa kwa matumizi ya baadaye kulingana na chaguo la mshiriki aliyechaguliwa hapo juu. .
- Ninathibitisha kuwa mshiriki alipewa nafasi ya kuuliza maswali juu ya hali na namna ya kuhifadhi sampuli, na maswali yote yaliyoulizwa na mshiriki yamejibiwa kwa usahihi na kwa kadiri ya uwezo wangu. Ninathibitisha kwamba mtu huyo hajalazimishwa kutoa idhini, na idhini hiyo imepewa kwa hiari na kwa hiari.

Nakala ya Fomu hii ya Ruhusa iliyoarifiwa imetolewa kwa mshiriki.

**Chapisha Jina la Mtafiti / mtu anayechukua idhini ________________________**

**Saini ya Mtafiti / mtu anayechukua idhini __________________________**

**Tarehe ___________________________**

**Siku / mwezi / mwaka**
